# Doxorubicin induces large-scale and differential H2A and H2B redistribution in live cells

**DOI:** 10.1371/journal.pone.0231223

**Published:** 2020-04-16

**Authors:** Péter Nánási, László Imre, Erfaneh Firouzi Niaki, Rosevalentine Bosire, Gábor Mocsár, Anett Türk-Mázló, Juan Ausio, Gábor Szabó

**Affiliations:** 1 Department of Biophysics and Cell Biology, Faculty of Medicine, University of Debrecen, Doctoral School of Molecular Cell and Immune Biology, Debrecen, Hungary; 2 Department of Immunology, Faculty of Medicine, University of Debrecen, Debrecen, Hungary; 3 Department of Biochemistry and Microbiology, University of Victoria, Victoria, British Columbia, Canada; Institute of Genetics and Molecular and Cellular Biology, FRANCE

## Abstract

We observed prominent effects of doxorubicin (Dox), an anthracycline widely used in anti-cancer therapy, on the aggregation and intracellular distribution of both partners of the H2A-H2B dimer, with marked differences between the two histones. Histone aggregation, assessed by Laser Scanning Cytometry via the retention of the aggregates in isolated nuclei, was observed in the case of H2A. The dominant effect of the anthracycline on H2B was its massive accumulation in the cytoplasm of the Jurkat leukemia cells concomitant with its disappearance from the nuclei, detected by confocal microscopy and mass spectrometry. A similar effect of the anthracycline was observed in primary human lymphoid cells, and also in monocyte-derived dendritic cells that harbor an unusually high amount of H2B in their cytoplasm even in the absence of Dox treatment. The nucleo-cytoplasmic translocation of H2B was not affected by inhibitors of major biochemical pathways or the nuclear export inhibitor leptomycin B, but it was completely diminished by PYR-41, an inhibitor with pleiotropic effects on protein degradation pathways. Dox and PYR-41 acted synergistically according to isobologram analyses of cytotoxicity. These large-scale effects were detected already at Dox concentrations that may be reached in the typical clinical settings, therefore they can contribute both to the anti-cancer mechanism and to the side-effects of this anthracycline.

## Introduction

Doxorubicin (Dox; also known as Adriamycin) is a widely used anthracycline anticancer drug which is applied in the treatment of various forms of leukemia and solid tumors, including T and B cell leukaemias, Hodgkin’s lymphoma, tumors of the bladder, breast, stomach and the lungs [1]. Overcoming its most common side effects, cardiotoxicity and treatment-related leukaemias, is a major challenge; both are rather specific for anthracyclines [2].

Dox is a pleiotropic drug having multiple targets. The main mechanisms of action include cell cycle block by topoisomerase II inhibition [3], inhibition of DNA and RNA synthesis [4], increased production of intracellular reactive oxygen species [5], and reorganization of F-actin [6]. Dox was shown to induce autophagy [7] and also to cause its dysregulation by inhibition of lysosomal acidification [8].

The DNA and/or chromatin-related effects may be explained by multitudes of molecular interactions: Anthracyclines intercalate between the neighboring base-pairs of the double-helix [9], bind free histones [10], can form anthracycline-DNA covalent adducts [11] and are able to destabilize G-quadruplex structures [12]. Intercalation is accompanied by the release of histones and eventually with eviction of the complete nucleosome [13–15]. All this is not surprising considering that it relaxes the natural twist of the DNA double helix by −27°/ intercalating molecule [16]. Intercalation also changes the DNA length and rigidity [17] and increases the melting point of the double-helix [18].

Anthracycline-induced nucleosome eviction is accompanied by de-repression of many genes [13] and by the generation of double-strand DNA breaks at active gene promoters by the torsion-based enhancement of nucleosome turnover [19]. Eviction of endogenous and GFP-tagged histones (H2A, H2B, H3 and H4) from chromatin occurs at ≥9 μM Dox, overlapping the peak concentrations of the drug observed in the plasma of patients undergoing Dox therapy [13]. Daunomycin, another anthracycline drug, also induces release of the H1 and H2B histones from the chromatin and aggregation of the latter in live cells at clinically relevant concentrations [20].

Antracycline induced chromatin aggregation was initially described using analytical ultracentrifuge analysis, equilibrium dialysis and circular dichroism [21]. The mechanism of aggregation was suggested to involve intercalation of the drug to the linker DNA causing unwinding of the double-helix, followed by the release of the H1 linker histones [20,22], what would result in an unfolded chromatin conformation and aggregation due to histone-DNA interfiber interactions [11,23]. Morphological changes were observed in the fluorescent microscope upon anthracycline treatment of cells harboring fluorescent protein-tagged histones that were interpreted in terms of chromatin aggregation [20]. Relocation of H1 to nucleoli was also observed in the same study [20]. The appearance of H1 in the cytoplasm of dendritic cells (DCs) upon activation [24] was also reported. Anthracycline-induced aggregation of H2A (rather than of chromatin) and the nucleo-cytoplasmic translocation of H2B, demonstrated herein in live cells, have not been described earlier to our knowledge.

We have developed a Laser Scanning Cytometry (LSC)-based method to quantitatively assess histone aggregation and studied redistribution of the evicted histones by confocal laser scanning microscopy (CLSM), as well as mass spectrometry (MS). We report here a global and differential aggregation/redistribution of the two members of the histone dimer upon Dox treatment of Jurkat and human peripheral blood mononuclear cells (hPBMCs), as well as in monocyte-derived DCs.

## Results

### Marked aggregation of H2A but not of H2B induced by Dox treatment

**Fig 1:**
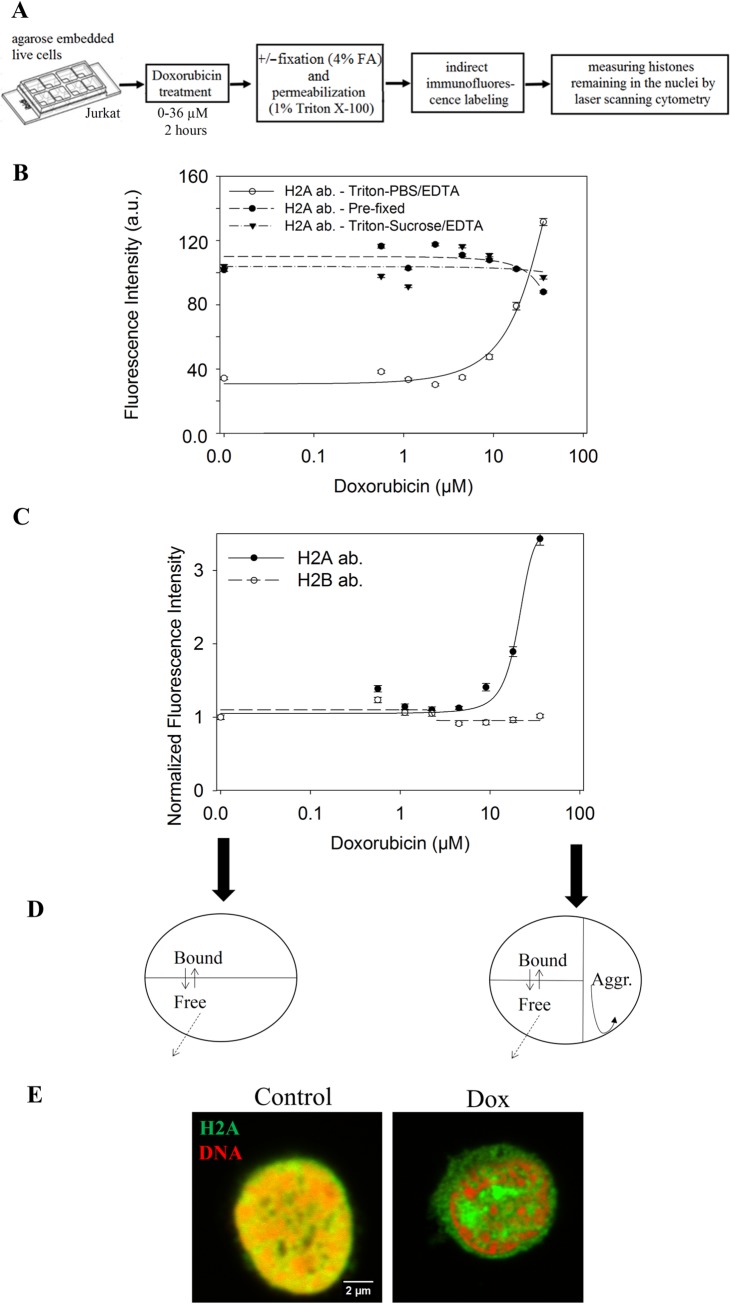
Marked aggregation of H2A, but not of H2B upon Dox treatment. (A) Cytometric assay of histone aggregation. Agarose-embedded live Jurkat cells were treated with Dox, then fixed and subsequently permeabilized („Pre-fixed”) or lyzed only. H2A or H2B were detected by indirect immunofluorescence and analyzed by LSC. G_1_ cells were gated based on the PI distribution histograms. (B) H2A levels after treatment with different concentrations of Dox, cells lyzed with 1% Triton X-100 in PBS/EDTA after Dox treatment (continuous line), cells pre-fixed and then permeabilized by the same lysing solution (dashed line), cells lyzed with 1% Triton X-100 in sucrose/EDTA (dash-dotted line). The symbols represent measured points, while the lines show the best fit as described in Materials and Methods. LSC settings (photomultiplier voltage gain and offset) were identical during the measurement of all the samples. (C) H2A (continuous line) and H2B (dashed line) levels after treatment with different concentrations of Dox. Fluorescence intensities were normalized to the intensity of untreated samples. In (B) and (C) error bars show SEM values, characterizing population heterogeneity, calculated for the G_1_ cell population in a representative experiment. LSC settings were as before. (D) Proposed scheme of Dox-induced aggregation dependent H2A retention. In the permeabilized nuclei, free H2A molecules diffuse out before immunofluorescence labeling, while aggregated and chromatin bound histones remain inside the nuclei. (E) Confocal microscopic images of cells treated with 36 μM Dox and of control cells. After fixation, H2A was detected by immunofluorescence (green) and DNA by PI staining (red). Instrument settings (laser power, photomultiplier voltage, gain, pixel dwell time) and all settings of the image analyses were identical for the compared samples of this panel. Single channel images and an image with amplified DNA signal are shown in S1 Fig.

We detected intranuclear aggregation of H2A, but not of H2B histones, in Jurkat cells after treatment with Dox, using a LSC-based procedure shown in Fig 1A. The agarose-embedded live Jurkat cells were treated with 0–36 μM Dox for 2 hrs, then either fixed (pre-fixation) and subsequently permeabilized by the Triton X-100-containing lysing solution, or treated with this solution without previous fixation (as described in Materials and Methods). Our interpretation of the aggregation dependent redistribution of H2A induced by Dox is shown in Fig 1D. Free histones, including those evicted by Dox, could diffuse out of the nuclei and were subsequently washed out, while aggregated and/or DNA-bound histones remained in the nuclei and were labeled by indirect immunofluorescence for subsequent analyses by LSC. The level of H2A in the nuclei of control cells was much lower than that of the pre-fixed samples, because the latter contain a pool of free histones readily diffusing out from the nuclei of the Dox-untreated cells. When sucrose rather than salt was used to set osmolarity, an old practice preserving nuclear morphology [25], the phenomenon was not detectable (Fig 1B). Sucrose, by increasing viscosity and thereby significantly decreasing diffusion, apparently facilitated rebinding of the histones evicted by Dox in the time interval prior to fixation and immunofluorescence labeling. After treatment of the cells with Dox, increased amounts of H2A remained in the nuclei obtained by Triton X-100/PBS-EDTA lysis. Retention was Dox concentration-dependent, starting below somewhat 9 μM and reaching the H2A levels of the pre-fixed control nuclei at 36 μM drug concentration. The rise in H2A level somewhat surpassed what was measured in pre-fixed cells most likely because of the decreased binding of the antibody to the formaldehyde-fixed antigens. In sharp contrast with H2A, no retention of H2B was detected in the assay when H2A and H2B were simultaneously measured (Fig 1C). Confocal microscopic images demonstrating the distribution of H2A retained in the nuclei after Dox treatment are shown in Fig 1E. The aggregated histones appeared to localize mostly to the space outside the trabeculate compartment of genomic DNA stained with propidium iodide (PI), corresponding to aggregated chromatin. No DNA could be detected within the aggregated H2A domains even at maximal amplification of the DNA signal (S1 Fig).

### Massive nucleo-cytoplasmic translocation of endogenous H2B, but not of H2A, after Dox treatment

**Fig 2:**
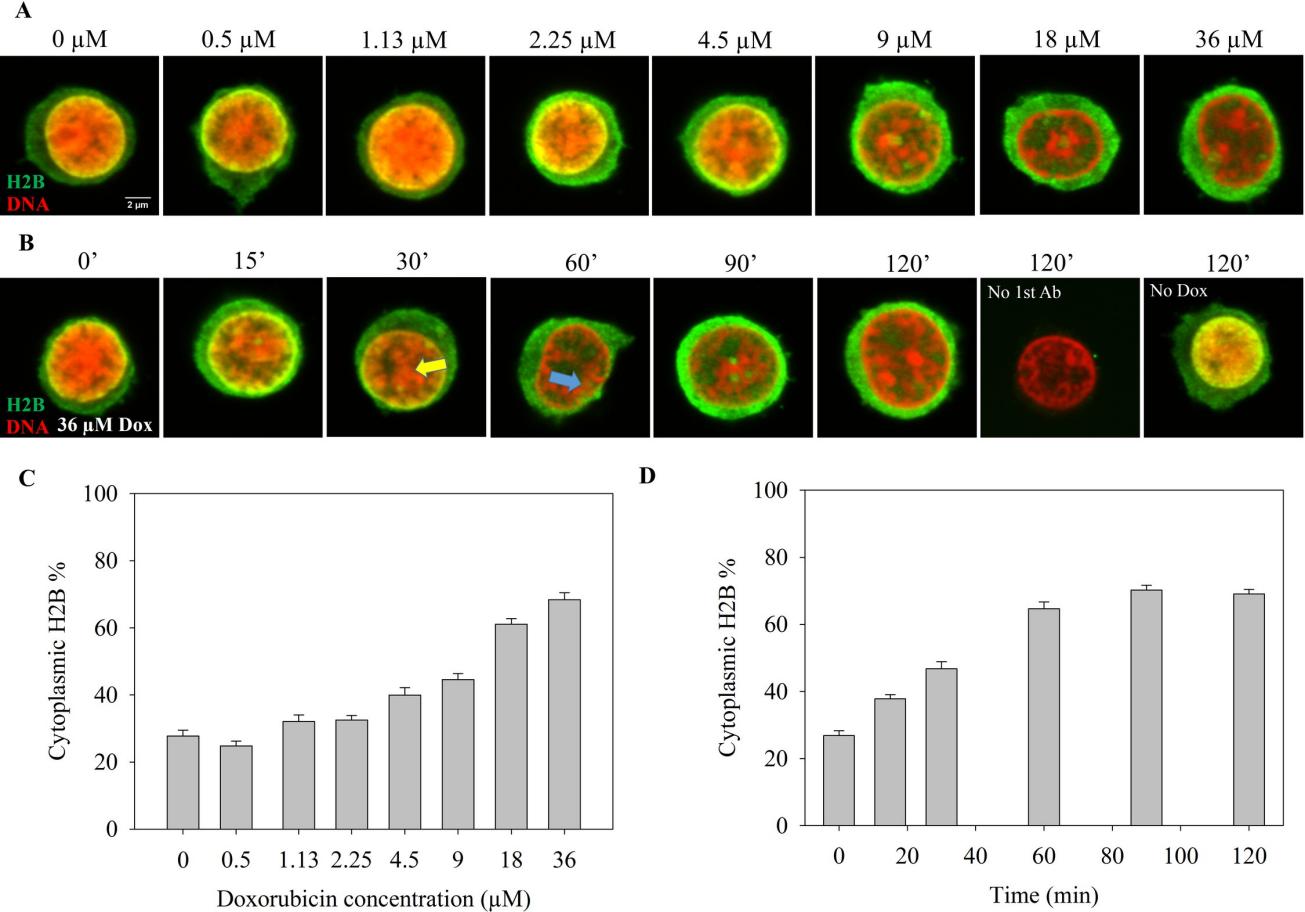
Nucleo-cytoplasmic translocation of endogenous H2B after Dox treatment. (A) Dox concentration dependence of H2B nucleo-cytoplasmic translocation. H2B was detected by immunofluorescence (green), DNA was stained with PI (red) in the fixed Jurkat cells. Representative confocal microscopic images are shown. For single channel images see S2 Fig. (B) Time dependence of Dox-induced H2B nucleo-cytoplasmic translocation. The yellow and blue arrows show depletion of H2B from the central and peripheral nuclear regions, respectively. Control cells incubated in the absence of Dox or labeled only with secondary antibody are also shown. Staining of H2B and of DNA were as in (A). Instrument settings (laser power, photomultiplier voltage, gain, pixel dwell time) and settings of image analyses performed by ImageJ were adjusted so as to detect also cytoplasmic H2B in the control cells and were identical for each compared sample of a particular experiment, both in the case of panel A and B. For single channel images see S2 Fig. (C), (D) Quantification of fluorescence microscopic images of panel A and B, respectively. The total cytoplasmic H2B immunofluorescence was determined as described in Materials and Methods and normalized to the total cellular immunofluorescence. Error bars represent SEM.

As compared to H2A, histone H2B behaved in a sharply different manner upon Dox treatment not only in terms of aggregation tendency (see Fig 1C), but also in its intracellular localization. As shown in Fig 2A–2D, H2B accumulated in the cytoplasm in a Dox concentration and time-dependent manner, forming a gradient with ~3x higher amount of the histone in the cytoplasmic compartment relative to the nucleus. H2B nucleo-cytoplasmic translocation, likely an active process in view of the gradient formed (Fig 2C), started at a Dox concentration as low as ~2 μM. Depletion of H2B from the center of the nucleus occurred already after 15–30 mins of treatment and propagated to the periphery concomitantly with a gradual elevation of H2B detected in the cytoplasm (Fig 2B and 2D). After 2 hrs of Dox treatment, the majority of H2B was depleted from the nuclei and accumulated in the cytoplasm.

**Fig 3:**
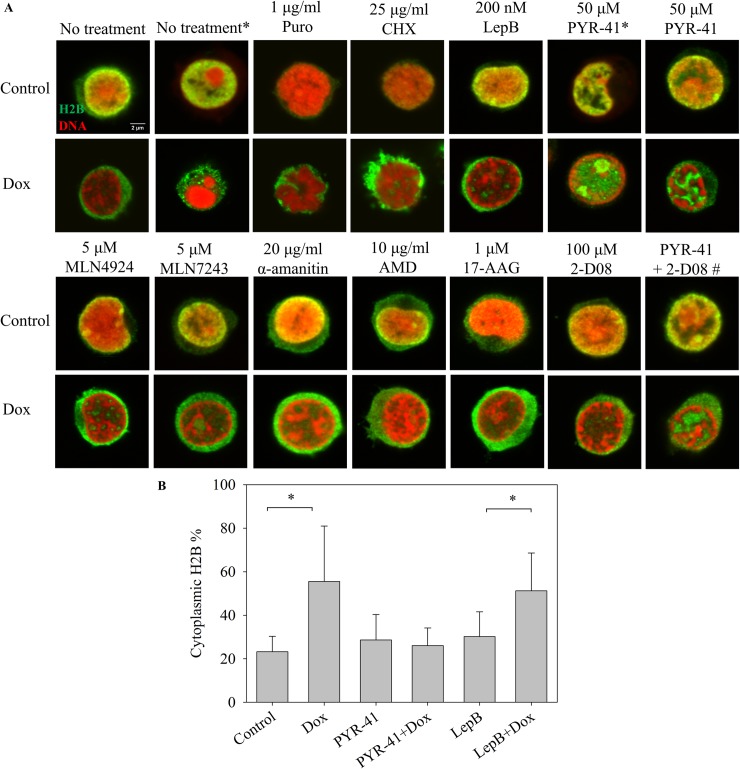
Effect of various inhibitors on Dox-induced H2B nucleo-cytoplasmic translocation. **(**A) The cells co-treated with the drug(s) indicated in the Figure and with 36 μM Dox for 2 hrs were fixed and stained as in Fig 1E. H2B was labeled with a monoclonal anti-H2B antibody, except for the samples marked with an asterisk (*) that were labeled with a polyclonal anti-H2B antibody (see Materials and Methods). # marks the sample treated with both 50 μM PYR-41 and 100 μM 2-D08 (upper image), or co-treated with the two inhibitors and 36 μM Dox (lower image). DNA was stained with PI. H2B immunofluorescence: green; PI: red. Representative confocal microscopic images are shown. Instrument settings and settings of image analyses performed by ImageJ were identical for each compared sample of a particular experiment. Single channel images are shown in S3 Fig. (B) Quantification of fluorescence microscopic images of panel A. The total cytoplasmic H2B immunofluorescence was determined as described in Materials and Methods and normalized to the total cellular immunofluorescence. Error bars represent SEM. * indicates significant difference based on two-tailed Student’s t-test (p<0.001).

To investigate the possible mechanisms of cytoplasmic H2B accumulation, treatment of the Jurkat cells with inhibitors of biochemical processes that could perhaps account for the cytoplasmic accumulation of H2B were performed (Fig 3). The possible role of an increased *de novo* protein synthesis in the accumulation of cytoplasmic H2B was excluded by blocking the synthetic process with puromycin or cycloheximide. Inhibition of CRM1 mediated nuclear export by leptomycin B failed to diminish the Dox-induced H2B translocation. The anthracycline-induced histone aggregates may be recognized by the cell as denatured proteins destined for elimination by degradation and/or export out of the nuclei [26]. Therefore, inhibitors affecting such pathways were also tested. H2B export could be strongly diminished by PYR-41, an inhibitor of diverse processes including E1-mediated ubiquitination [27]. After co-treatment with Dox and PYR-41, H2B showed a characteristic nuclear localization pattern with spatial separation of the histone and the DNA-containing chromatin, reminiscent of the segregation of H2A from chromatin after Dox treatment alone. Dox-induced H2B export could not be reduced by another ubiquitination inhibitor MLN7243, the NEDDylation inhibitor MLN4924 or the SUMOylation inhibitor 2-D08. Co-treatment with 2-D08 partially attenuated the effect of PYR-41 on Dox-induced H2B translocation, leading to a moderate cytoplasmic accumulation of H2B upon Dox treatment. Neither inhibitors of transcription (α-amanitin or actinomycin D), nor an inhibitor of Hsp90 (17-AAG) were able to revert the Dox-induced H2B export. H2B cytoplasmic accumulation was detected using two different (monoclonal vs. polyclonal) anti-H2B antibodies. Both commercially available antibodies are extensively used to visualize H2B with high specificity and sensitivity [28–30]. Nonspecific binding of the secondary antibody was ruled out by incubating the cells with the secondary antibody only (see Fig 2).

**Fig 4:**
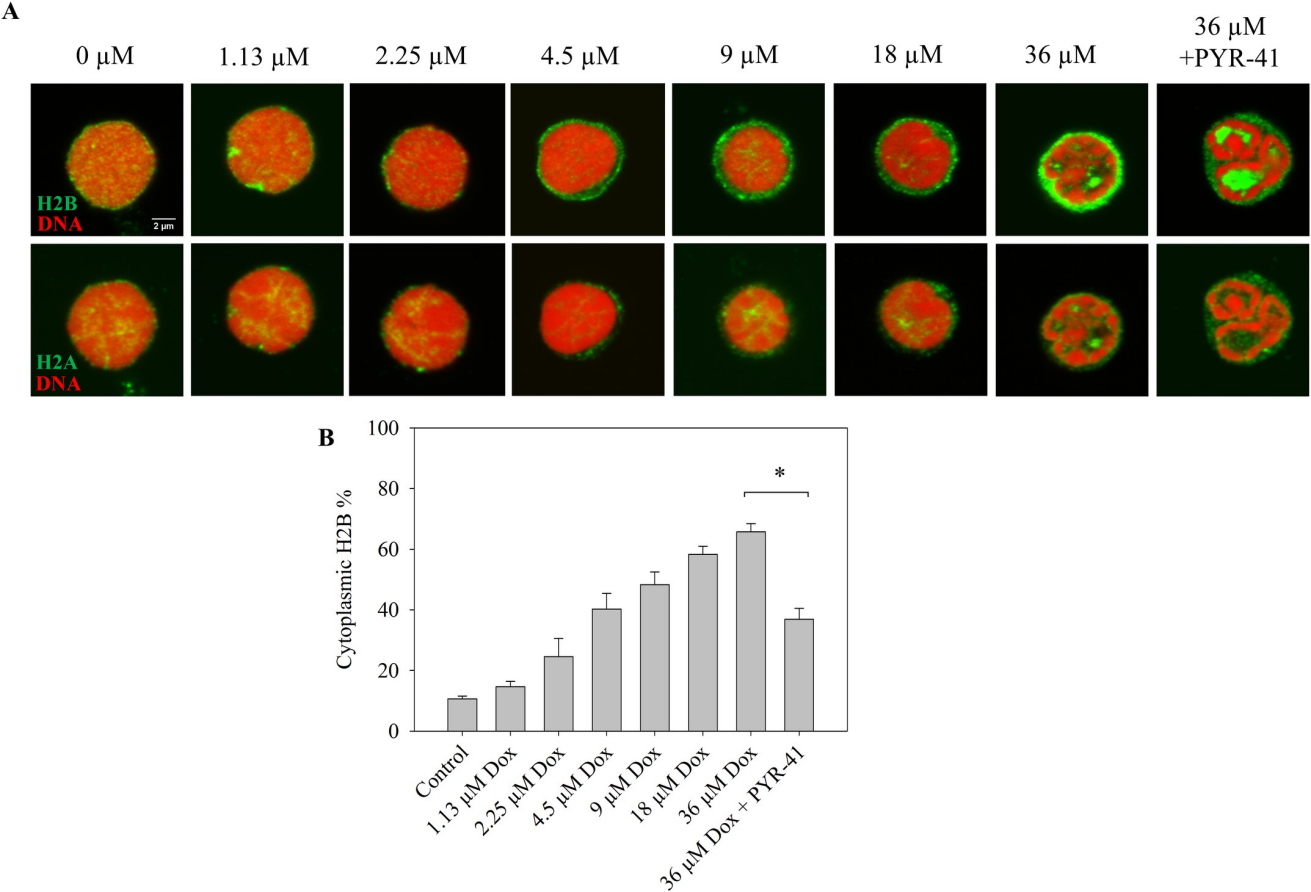
Nucleo-cytoplasmic translocation of H2B but not of H2A, and histone aggregation after Dox treatment of hPBMCs. (A) Upper row: Dox concentration dependence of H2B nucleo-cytoplasmic translocation. Lower row: H2A immunofluorescence in the same cells that are shown in the upper row. The cells were treated with Dox alone or with Dox and 50 μM PYR-41 together for 2 hrs. DNA was stained with PI. The colors are as in the previous Figures. Representative confocal microscopic images are shown. Instrument settings and settings of image analyses performed by ImageJ were identical for each compared sample of a particular experiment. Single channel images are included in S4 Fig. (B) Quantification of fluorescence microscopic images of panel A. The total cytoplasmic H2B immunofluorescence was determined as described in Materials and Methods and normalized to the total cellular immunofluorescence. Error bars represent SEM.* indicates significant difference based on the two-tailed Student’s t-test (p<0.001).

We investigated cytoplasmic H2B relocation also in primary human lymphoid cells. As Fig 4 demonstrates, Dox treatment elicited cytoplasmic relocation of H2B in human peripheral blood mononuclear cells (hPBMCs). In further similarity with the effects demonstrated in Jurkat cells, Dox caused no translocation of H2A in hPBMC nuclei but induced its segregation from chromatin that exhibited a trabeculate staining pattern. H2B translocation was overruled by PYR-41 treatment also in these cells, when the histone accumulated between the DNA-containing chromatin domains.

**Fig 5:**
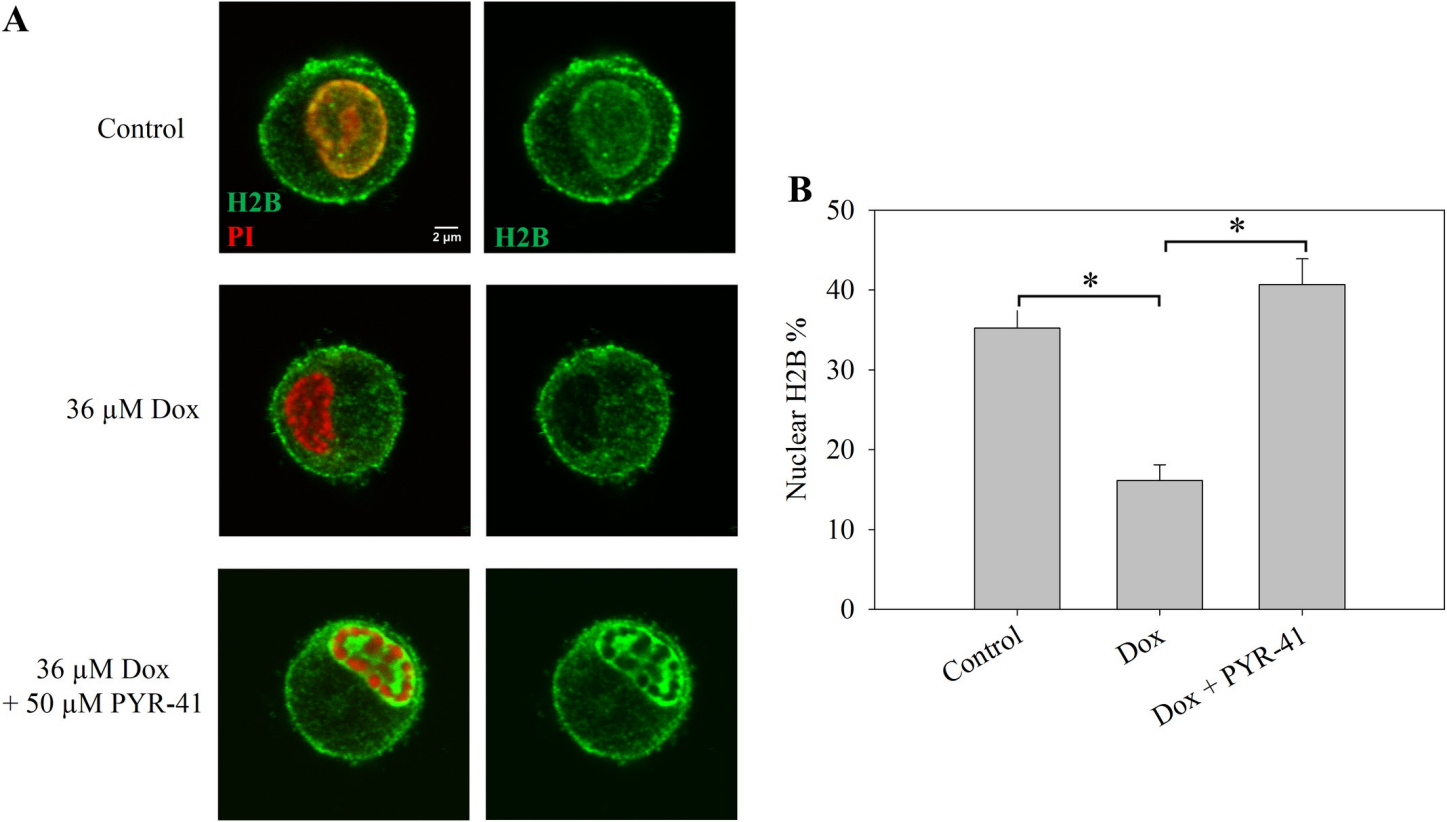
Effect of Dox and PYR-41 on the levels of H2B histones detected in the cytoplasm of DCs. (A) Cells treated with the drug(s) indicated in the Figure for 2 hrs were fixed and labeled with a monoclonal anti-H2B antibody (green) and the DNA was stained by PI (red). Representative confocal microscopic images are shown. Instrument settings and settings of image analyses performed by ImageJ were identical for each compared sample of a particular experiment. More images are shown in S9 Fig. (B) Quantification of fluorescence microscopic images of panel A. The total nuclear H2B immunofluorescence was determined as described in Materials and Methods and normalized to the total cellular immunofluorescence. Error bars represent SEM.* indicates significant difference based on the two-tailed Student’s t-test (p<0.001).

In view of the involvement of extranuclear H2B in innate immunity [31–34], we tested the effect of Dox on the distribution of this histone in monocyte-derived human DCs. Similarly to the other cells studied, Dox triggered a near-complete eviction of H2B from the nuclei, what was reverted by PYR-41, as shown in Fig 5. H2B was present in the cytoplasm of these cells at a surprisingly high level even in the absence of any treatment.

The Dox-induced nucleo-cytoplasmic H2B export was confirmed using mass spectrometric (MS) analyzes of supernatants of the cytoplasmic cell lysates, performed in parallel with the microscopic assessment of H2B remaining in the nuclei (S8 Fig). MS analysis showed an elevated H2B content in the supernatant of Dox-treated lyzed cells, simultaneously with the vanishing of the histone from the nuclei, as compared to the untreated control.

The MS analyses also showed that a number of other histones (Table 1) and nuclear proteins (Table 2) were released from the nuclei by Dox. Under these experimental conditions, *i*.*e*. upon lysis of the cytoplasmic content by Triton X-100 and following centrifugation, increased amounts of other histones, prominently H1.2, was also detected in the supernatant of Dox-treated cells. However, only the appearance of H2B in the supernatant was PYR-41-sensitive. A series of non-histone proteins also exhibited increased elution from the nuclei upon Dox treatment under these conditions (Table 2). Both the magnitude of the Dox-induced protein release from the nucleus and their PYR-41-sensitivity varied greatly among the non-histone nuclear proteins as well.

**Table 1:** Histones detected by MS in the supernatant of Dox-treated, Dox + PYR-41-treated and untreated samples.

Histone	Protein name	Control	Dox	Dox + PYR-41	Fold change (Dox/Control)	Fold change (Dox + PYR-41/Control)
H1.2	Histone H1.2	7.14E-04	5.24E-03	9.80E-03	7.34	13.73
H1.5	Histone H1.5	1.44E-03	3.00E-03	4.08E-03	2.09	2.84
H2A	Histone H2A type 1-J	1.39E-03	4.37E-03	2.63E-03	3.15	1.90
H2B	HIST1H2BC	2.06E-03	4.17E-03	2.44E-04	2.02	0.12
H4	HIST1H4A	1.64E-03	3.95E-03	1.98E-03	2.41	1.21

The values represent ratios of the histone specific molecular fragments relative to the internal standard. Fold changes induced by Dox and by Dox + PYR-41 were calculated as the ratio of proteins detected in the supernatants of drug-treated and control samples. Dox-treated cells: Dox; Dox+PYR-41-treated cells: Dox+PYR-41; untreated cells: Control.

**Table 2:** Examples of non-histone proteins detected by MS in the supernatants of Dox-treated, Dox + PYR-41 co-treated and control samples.

Protein function	Increased fraction	Examples	Fold change (Dox)	Fold change (Dox + PYR-41)
Chromatin architecture	9/9	*Isoform 2 of Nucleosome assembly protein 1-like 1*	6.70	2.81
		Regulator of chromosome condensation 2, isoform CRA_a	4.42	3.79
		Isoform 2 of Histone deacetylase 2	1.68	0.79
		Histone acetyltransferase type B catalytic subunit	6.10	4.52
		Lamina-associated polypeptide 2, isoforms beta/gamma	2.80	2.60
Transcription / RNP / Splicing	32/34	*U5 small nuclear ribonucleoprotein 200 kDa*	*3*.*32*	*0*.*54*
		Heterogeneous nuclear ribonucleoprotein K, isoform CRA_d	2.39	1.87
		Pre-mRNA-processing factor 19	5.96	3.39
		RNA transcription, translation and transport factor protein	2.01	1.27
		Isoform 3 of 60 kDa SS-A/Ro ribonucleoprotein	2.36	2.08
Replication / Repair	20/20	*RuvB-like helicase (Fragment)*	*3*.*11*	*1*.*23*
		DNA helicase	2.28	2.42
		Poly [ADP-ribose] polymerase 1	2.70	2.07
		DNA-dependent protein kinase catalytic subunit	2.83	1.41
		DNA replication licensing factor MCM6	2.72	2.84
Nuclear Import / Export	8/8	*Importin-7*	*2*.*78*	*0*.*52*
		GTP-binding nuclear protein Ran	2.98	2.46
		Importin-5	2.35	2.24
		Importin subunit beta-1 OS	3.19	3.55
		Exportin-1	1.36	1.12
Ubiquitination / Proteasome / SUMOylation	32/34	*Proteasome subunit alpha type*	*2*.*72*	*0*.*63*
		SUMO-activating enzyme subunit 2	2.52	2.40
		SUMO-activating enzyme subunit 1	1.60	2.26
		Polyubiquitin-B (Fragment)	3.54	4.25
		Proteasome activator complex subunit 2	2.00	2.11
Heat Shock Proteins	4/5	Heat shock 60kDa protein 1 (Chaperonin), isoform CRA_a	2.25	2.53
		Heat shock protein HSP 90-beta	2.51	2.58
		Heat shock 70 kDa protein 4	2.71	2.71
		Isoform Beta of Heat shock protein 105 kDa	2.34	2.16

Fold change was calculated as in Table 1. Increased fraction in the functional categories designates the ratio of the number of different proteins with elevated levels in the supernatant (fold change (Dox) > 1) and the total number of identified proteins in that functional group. Proteins exhibiting reversal of the Dox-effect by PYR-41 are shown in italics.

**Fig 6:**
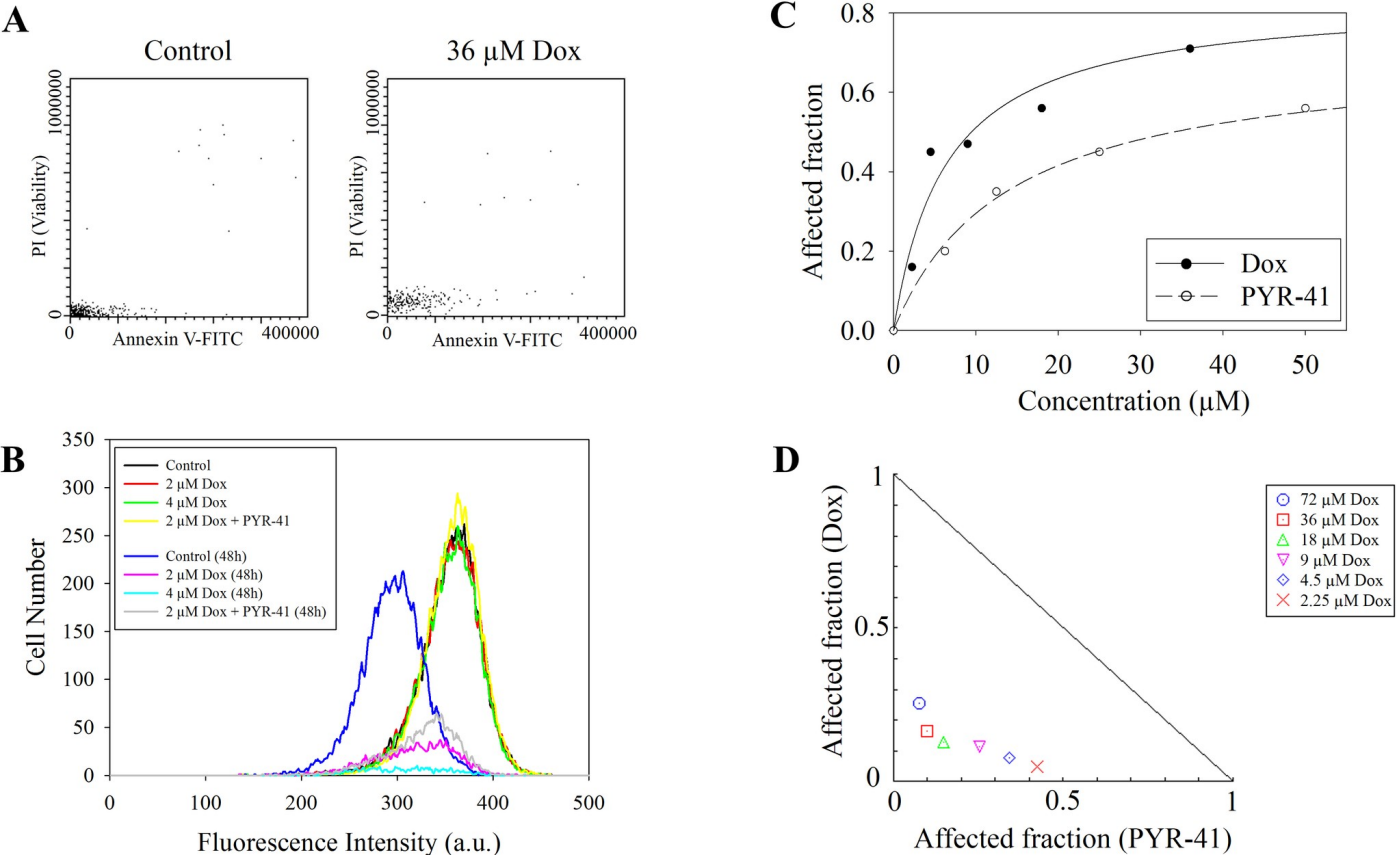
Early and late effects of Dox treatment on the viability of Jurkat cells. (A) Viability assessed immediately after 2 hrs of treatment with 36 μM Dox as compared to that of the control cells. The scatterplots show Annexin V-FITC and PI staining of G_1_ gated cells. (B) CellVue dye-dilution cell proliferation assay of Dox and Dox + 6 μM PYR-41 co-treated Jurkat cells (see Materials and Methods). Far red fluorescence intensity distributions were recorded at 2 hrs and at 48 hrs after addition of the drugs. PI positive cells were excluded from the analyses. (C) Dose-Effect Curve for Dox and PYR-41 based on resazurin assay performed 24 hrs after the 2 hrs Dox treatment. (D) Normalized Isobologram of Dox + PYR-41 co-treatment, using 50μM PYR-41 and Dox applied in a concentration range of 2.25–72 μM. In this representation the data points below or above the line indicate synergistic or antagonistic drug interactions, respectively; see Materials and Methods.

We investigated the possibility if the cytoplasmic presence of H2B is the result of decreased cell viability. As Fig 6A demonstrates, the Dox-treated cells showed neither PI uptake nor an increased Annexin V binding after the 2 hrs of Dox treatment. (The red signal was somewhat upshifted in the Dox-treated samples since the fluorescence emission of the drug partially overlaps with the emission spectrum of PI using the filter set described in Materials and Methods.) The impression that histone aggregation and relocation were not the result of cytotoxicity manifesting at the end of 2 hrs of Dox treatment was also confirmed by the fact that the degree of H2A retention was not sensitive to the caspase inhibitor Z-VAD-FMK (S5 Fig). Dox concentration dependent toxicity was detected by the resazurin assay 24 hrs after Dox treatment (see Fig 6C). According to the normalized isobologram in Fig 6D, Dox and PYR-41 (the agent that reversed Dox-induced H2B translocation; see Fig 3) were cytotoxic in a synergistic manner, suggesting that H2B accumulation in the cytoplasm may not significantly contribute to long-term toxicity. Indeed, cell proliferation was inhibited by 2 hrs of Dox treatment also in the case of co-treatment with PYR-41 as measured by the CellVue dye-dilution assay after 2 days, based on the fact that the corresponding distribution histograms have similar medians (Fig 6B; magenta and grey lines).

## Discussion

Dox treatment induced a marked nuclear aggregation of H2A, as opposed to H2B, at therapeutically relevant concentrations (Fig 1B and 1C). Chromatin aggregation upon anthracycline treatment has been described earlier in *in vitro* [11,23] and *in vivo* [20] systems. This is the first report to our knowledge describing such phenomena involving histones alone. Histones are known to be evicted from the chromatin by Dox in an intercalator concentration dependent manner (see [13,15,20,23]). Through the spectacles of confocal microscopy the majority of H2A molecules detected by immunofluorescence becomes topologically separated from the DNA-containing regions upon treatment with >9 μM Dox and gets trapped inside the nuclei as measured in our LSC-based assay (Fig 1, S1 Fig). Thus, the nuclear consequences of Dox treatment are the composite result of eviction and aggregation rather than of eviction alone.

H2B also appeared to be segregated from the DNA-containing chromatin compartment when its exodus to the cytoplasm was prevented by PYR-41 co-treatment (Figs 3 and 4). Since aggregation was not detectable in our LSC-assay (S6 Fig), the composition, size and/or stability of the aggregates (formed in the presence of PYR-41) may be different from that of the H2A aggregates. The fact that H2A and H2B can be separated by HPLC, implying a difference in the overall hydrophobicity of the two histones [35], is in line with their differential aggregation tendencies upon Dox treatment, what may be due to different amounts of the drug bound to the two histones. The two members of the dimer can be released from the tetrasomes independently from each other (see S13 Fig of ref. [15]), so they could also be independently affected by Dox treatment. In the absence of PYR-41 H2B probably escapes aggregation because of its disappearance from the nuclei.

Dox caused a massive increase of cytoplasmic H2B, but not of H2A levels (Figs 3 and 4). The cytoplasmic accumulation of H2B upon Dox treatment is a phenomenon not described before for any of the histones to our knowledge. This is to be taken into account among the responses of cells to Dox treatment in view of the small concentration of Dox required for the effect, what was detected in Jurkat cells, hPBMCs as well as DCs. The fact that H2B accumulated in a trabecular pattern outside the DNA-containing compartment in the samples co-treated with PYR-41 (Figs 3 and 4) shows that the dissociation of the histone from chromatin induced by Dox was not affected, i.e. just the export of H2B was inhibited by PYR-41. The concomitant accumulation of H2B in the cytoplasm was detected by immunofluorescence, using either a monoclonal or a polyclonal antibody (Fig 2C and 2D), and it was observed in the MS experiment (S8 Fig) as well, where it also exhibited PYR-41 sensitivity. The fact that H2B could be detected by the same antibodies in the nucleus in PYR-41 co-treated cells (Fig 3) is also taken as evidence for the specificity of immunofluorescence detection. Although anthracycline-induced intranuclear (nucleolar) H1 relocation was readily detected using GFP-tagged H1 expressor cells [20], we observed no nucleo-cytoplasmic export of H2B-GFP (S7 Fig). The difference between the re-location of H2B, as detected by immunofluorescence, and the lack of it in the case of H2B-GFP, was clearly demonstrated when both entities were visualized at the same time (S7 Fig). The interference of the GFP tag with nucleo-cytoplasmic translocation is in line with the involvement of a specific transport mechanism and may not be surprising in view of the fact that even a Flag tag can have profound effects on the molecular interactions involving H2B [36].

Highly reduced intranuclear H2B content along with highly increased cytoplasmic H2B levels upon Dox treatment (Figs 2C, 2D, 4B and 5) can be explained by the presence of an active export mechanism and intact nuclear membranes, the latter serving as a barrier impeding H2B nuclear re-entry. In contrast with its complete reversal effected by PYR-41, cytoplasmic accumulation of H2B was not significantly altered by inhibiting CRM1-dependent nuclear export, or applying inhibitors of ubiquitination, NEDDylation, SUMOylation, transcription, protein synthesis or Hsp90 function (Fig 3). PYR-41 acts mainly as an inhibitor of the ubiquitin-activating enzyme (E1), with little or no effect on the ubiquitin-conjugating enzyme (E2) [27]. However, the agent was also shown to enhance total SUMOylation in cells [27]. Induction of non-specific protein cross-linking may also contribute to its toxicity [37]. Furthermore, it seems to be also a deubiquitinase (DUB) inhibitor [37]. The other inhibitor of E1-mediated ubiquitination tested herein did not reproduce the effect of PYR-41, and the combined treatment with 2-D08 SUMOylation inhibitor mitigated the PYR-41-elicited reversal of cytoplasmic H2B accumulation following Dox treatment (when H2B was present at high levels both in the cytoplasm and in the nucleus; see Fig 3). Thus we assume that H2B export may be connected to the biochemical circuitry controlling protein degradation via the ubiquitin-like posttranslational modification, SUMO. The main nuclear export pathway is CRM1 (also known as exportin-1 or XPO1) dependent, which can be inhibited by leptomycin B that alkylates and inhibits the CRM1 protein [38]. The insensitivity of H2B translocation to even high concentrations of this agent argues against the involvement of this mechanism. Several CRM1 independent nuclear export pathways have been described, including the transport of mature mRNAs [39,40], of poly(A)^+^ RNAs [41] and RNA helicases [42]. Biochemical processes involving SUMOylation are important determinants of nuclear export of mRNAs [43] and proteins [44]. H2B translocation from the nucleus to the cytoplasm further increases the plethora of molecular events that are to be considered as components of Dox toxicity.

The likely involvement of protein degradation-related pathways in Dox-induced H2B nucleo-cytoplasmic export (Figs 3 and 4) raises the possibility that the phenomenon may have relevance in connection with the following published observations: Enhanced protein degradation may play an important role in the acute cardiotoxicity of Dox therapy [45]; down-regulation of UBC9, an E2-conjugating enzyme that is required for SUMOylation, increases the sensitivity of hepatocellular carcinoma to Dox [46]; the SUMO pathway is a major determinant of Dox cytotoxicity in yeast [47]; down-regulation of histone H2A and H2B pathways is associated with anthracycline sensitivity in breast cancer [48]. It will be interesting to elucidate the exact relationship between the protein degradation-related pathways and H2B cytoplasmic accumulation to investigate the above possible links.

The nucleo-cytoplasmic translocation of H2B induced by Dox may be connected to H2B export occurring in certain physiological and pathological circumstances. Instances of extra-nuclear location of histones have been documented [49,50]. Extracellular histones can function as microbicidal proteins [34], on the other hand histones released in sepsis contribute to endothelial dysfunction [51]. Apoptosis unrelated nucleo-cytoplasmic translocation of H1 histone was detected in HeLa cells [52], and extrachromosomal H2B is known to mediate innate antiviral immune responses [31,32,53]. Remarkably, H2B in complex with gamma-interferon-inducible protein 16 was shown to be present in the cytoplasm during Epstein-Barr Virus and Herpes Simplex Virus-1 infection in non-apoptotic cells [33]. Dox treatment evoked a PYR-41 sensitive nucleo-cytoplasmic translocation of H2B also in DCs, similarly to Jurkat and hPBMCs, resulting in significantly decreased intranuclear H2B levels. Intriguingly, DCs exhibit high cytoplasmic levels of H2B in the absence of any treatment (Fig 5), especially in plasma membrane proximal areas. Since macrophages and DCs are the frontline cells of innate immunity [54], it is possible that the elevated cytoplasmic H2B levels in DCs are required for pattern recognition (Fig 5) [55]. The fact that the cytoplasmic levels of H2B can be readily increased by treatment with >2 μM Dox (Fig 2) raises the intriguing possibility that the H2B-related pathways of antiviral and antimicrobial immune response may be boosted by Dox treatment.

As Tables 1 and 2 show, Dox treatment facilitated the release of a wide variety of nuclear proteins. The proteins exhibiting enhanced release from the nucleus upon Dox treatment (Table 2) are very diverse, suggesting that the entire nuclear structure is affected by the drug. This Dox-induced effect is apparently superimposed on a background of non-specific release of nuclear proteins likely explained by the conditions required in the MS experiment. These include non-ionic detergent treatment as well as centrifugation of the nuclei with concomitant mechanical stress, i.e. steps that are absent when agarose-embedded live cells are treated with the anthracycline, fixed with 4% FA and processed for immunofluorescence detection. This experimental scenario apparently facilitated the discharge of proteins from the nucleus, demonstrated by the fact that Dox-aggregated H2A, shown not to be translocated to the cytoplasm under the less-perturbed conditions required by immunofluorescence detection (Figs 3, 4 and 5), also appeared in the supernatant of the Dox-treated samples (S8 Fig). Importantly, the appearance of H2A in the supernatant was much less PYR-41 sensitive than that of H2B in these conditions (Table 1). Destabilization of various other histones and an array of proteins involved in the organization of chromatin architecture, in transcription, splicing, replication, repair, nuclear export and import, in heat shock and those involved in protein degradation was observed upon Dox treatment, some of which also exhibited PYR-41-sensitive response to the drug (Tables 1 and 2). These Dox-induced perturbations of intermolecular interactions in the nucleus may all contribute to the effects and side-effects of anthracyclines observed in the clinical practice.

The fact that there was no toxicity detected at 2 hrs after addition of Dox (Fig 6A), shows that redistribution of the histones occurs in live cells. H2A aggregation ensues at ≥10 μM Dox, giving rise to ~50% toxicity at 24 hrs (Fig 6C) and an impact on cell proliferation detectable after 48 hrs (Fig 6B), i.e. aggregation measured at 2 hrs may be predictive of long-term toxicity and could be exploited as its convenient indicator. H2B translocation may be an even more sensitive predictor as it can be observed already at ~2 μM Dox concentration, resulting in ~15–20% cytotoxicity at 24 hrs.

We speculate that the nucleo-cytoplasmic export of H2B, which is impeded by PYR-41, may be a protective pathway rescuing cells from the toxic effect of intranuclear histone aggregation. This scenario appears to be supported by the fact that co-treatment with PYR-41 and Dox exhibited a strongly synergistic cytotoxic effect, not merely additivity (Fig 6D). The observed synergy raises the possibility that combined administration of Dox and PYR-41 may be exploited in cancer chemotherapy, since PYR-41 is already considered as a potential anticancer agent [27]. Interestingly, PYR-41 also mitigates lung injury in sepsis by targeting the NF-κB pathway involved in the inflammatory response to circulating histones in septic conditions [51,56].

The reported range of peak plasma concentrations of Dox is rather wide: values between 0.5 and 9 μM have been reported [13,57–60]. Therefore, the Dox concentration range used in our experiments and the peak plasma concentrations in clinical settings could overlap. The cytotoxicity of many DNA-binding small molecules seems to correlate more with their ability to cause chromatin damage than with DNA damage [61]. Our observations expand the list of molecular changes supporting such a scenario, raising the possibility that the severe perturbations in H2A and H2B intracellular and intranuclear localization observed may be of interest also from the medical point-of-view, regarding both the effects and side-effects of anthracycline treatment. It will be intriguing to test if the novel phenomenon of Dox-induced H2B nucleo-cytoplasmic export may be exploited to boost or modulate immune response involving extrachromosomal H2B [31,32,53]. Aggregation of H2A may be a manifestation of the general protein aggregating effect of Dox, detected also in yeast [62] and considered to significantly contribute to the cardiotoxicity of this agent [63]. The LSC-based assay of H2A aggregation described herein may find its application also in studies addressing various other cell biological conditions involving protein aggregation in the nucleus.

## Materials and methods

### Cell culture

Jurkat cells were grown in T150 tissue culture flasks (Corning Glass Works, Corning, NY) using RPMI-1640 (Gibco, Grand Island, NY) and 10% fetal bovine serum. The cultures contained penicillin (100 μg/ml), streptomycin (0.25 μg/ml), and glutamine to a final concentration of 2 mM.

#### hPBMCs and human monocyte-derived DC cultures

Leukocyte-enriched buffy coats were obtained from healthy blood donors drawn at the Regional Blood Center of the Hungarian National Blood Transfusion Service (Debrecen, Hungary) in accordance with the written approval of the Director of the National Blood Transfusion Service of the University of Debrecen, Faculty of Medicine (Hungary) and from the Regional and Institutional Research Ethical Committee of the University of Debrecen. Written, informed consent was obtained from the blood donors prior to blood donation, their data were processed and stored according to the directives of the European Union. hPBMCs were separated by a standard density gradient centrifugation with Ficoll-Paque Plus (Amersham Biosciences, Uppsala, Sweden). Monocytes were purified from hPBMCs by positive selection using immunomagnetic cell separation and anti-CD14 microbeads, according to the manufacturer’s instruction (MiltenyiBiotec, Bergisch Gladbach, Germany). After separation on a VarioMACS magnet, 96–99% of the cells were shown to be CD14^+^ monocytes, as measured by flow cytometry. Isolated monocytes were plated at 1 x 10^6^ cell/ml concentration in AIM-V medium (Gibco, Paisley, Scotland) containing L-glutamine and supplemented by 1% Gentamicin/Streptomycin solution (Hyclone, South Logan, Utah) in the presence of 100 ng/ml IL-4 (Peprotech EC, London, UK) and 80 ng/ml GM-CSF (Gentaur Molecular Products, Brussels, Belgium) added on day 0 and 2. Monocytes were cultured for five days in 24-well tissue culture plates.

### Embedding live cells into low melting point agarose

Embedding was carried out according to Imre et al. [15]. Briefly, the wells of 8-well chambers (Ibidi, Martinsried, Germany) were coated with 1% (m/v) low melting point (LMP) Agarose. The cell suspension containing 6 x 10^6^ cells/ml of PBS was mixed with 1% LMP agarose diluted in 1 x PBS at 37°C, and the cell-agarose suspension was dispensed in the middle of the wells. After polymerization of the agarose on ice, complete culture medium was added to each well.

### Sample preparation for mass spectrometry

Lysis of the 36 μM Dox-treated and control cells was by mixing 0.8 ml of cell suspension containing 2 x 10^7^ cells/ml with 0.2 ml ice cold PBS containing 1% Triton X-100 on ice. After incubation on ice for 10 minutes, 14 ml of PBS was added in order to terminate lysis, then the nuclei were centrifuged at 550 g for 5 minutes. The supernatant was concentrated using Amicon Ultra 0.5 ml Centrifugal Filters (EMD Millipore, Darmstadt, Germany), then sent for Mass Spectrometry. The nuclei were fixed by incubating the pellet in freshly prepared 4% formaldehyde dissolved in PBS/EDTA, for 10 minutes, then embedded into agarose in 8-well chambers (Ibidi, Martinsried, Germany) as described above.

Protein concentration of the samples was determined by the Bradford method. Samples were purified on a 5% SDS-polyacrylamide gel using 80V current for 15 minutes. The proteins were stained with Coomassie dye. The proteins were excised from the gel and subjected to in-gel trypsin digestion. Reduction was performed using 20 mM dithiothreitol for one hour at 56°C followed by alkylation with 55 mM iodoacetamide for 45 minutes. Overnight trypsin digestion was carried out using stabilized MS grade TPCK-treated bovine trypsin (ABSciex) at 37°C. Thereafter the digested peptides were extracted and lyophilized. The peptides were re-dissolved in 10μl 1% formic acid before LC-MS/MS analysis. Prior to LC-MS analysis the samples were spiked with equal amount of indexed retention time (iRT) peptide mixtures (Biognosys) and the samples were analyzed in duplicates.

### LC-MS analysis

Before mass spectrometric analyzes, peptides were separated on a 180 minute water/acetonitrile gradient using an Easy nLC 1200 nano UPLC (Thermo Scientific, Waltham, MA, USA). The peptide mixture was desalted on an Acclaim PepMap 100 C18 trap column (20 x 75 μm, 3 μm particle size, 100 Å pore size, Thermo Scientific, Waltham, MA, USA), followed by separation on Acclaim PepMap RSLC C18 analytical columns (150 mm x 50 μm 2 μm particle size, 100 Å pore size, Thermo Scientific, Waltham, MA, USA). The peptides were separated using a gradient of 5–7% solvent B over 5 minutes, followed by a rinse to 15% of solvent B over 50 minutes, and then to 35% solvent B over 60 minutes. Thereafter solvent B was increased to 40% over 28 minutes and to 85% over 5 minutes, followed by a 10 minutes rinse to 85% of solvent B, after which the system returned to 5% solvent B in 1 minute for a 16 minutes hold-on. Solvent A was 0.1% formic acid in LC water; solvent B was 95% acetonitrile containing 0.1% formic acid. The flow rate was set to 300 nl/min.

Data-dependent analyzes were carried out on an Orbitrap Fusion mass spectrometer (Thermo Scientific, Waltham, MA, USA). The 14 most abundant multiply charged ions were selected from each survey MS scan using a scan range of 350–1600 m/z for MS/MS analyzes (Orbitrap analyzer resolution: 60000, AGC target: 4.0e5, acquired in profile mode). CID fragmentation was performed in the linear ion trap with 35% normalized collision energy (AGC target: 2.0e3, acquired in centroid mode). Dynamic exclusion was enabled during the cycles (exclusion time: 45 seconds).

### Mass spectrometry data analysis

The acquired LC-MS/MS data were used for protein identification and quantification with the help of MaxQuant 1.6.2.10 software [64] searching against the Human SwissProt database (release: 2018.08, 558125 sequence entries). Cys carbamidomethylation was set as fixed modification, Met oxidation and N-terminal acetylation were set as variable modifications. Maximum 2 missed cleavage sites were allowed. Proteins were accepted with at least 3 identified peptides using 1% FDR criteria. Label-free protein quantification was performed with the LFQ algorithm of the MaxQuant software [65]. For data evaluation, the LFQ values of the identified proteins were normalized to the LFQ values of the iRT mixture, relative amount of proteins was calculated and indicated in Table 1.

The H2A and H2B histones remaining in the nuclei were labeled by immunofluorescence. These intensities and the proteins levels measured in the supernatant by mass spectrometry were compared on the same scale (see S8 Fig), based on the assumptions expressed by the equations:

$$H2A‐CTRL_{\left(n\right)}+a\;*\;H2A‐CTRL_{\left(sn\right)}=H2A‐CTRL_{\left(t\right)}$$

$$H2A‐Dox_{\left(n\right)}+a\;*\;H2A‐Dox_{\left(sn\right)}=H2A‐Dox_{\left(t\right)}$$

$$H2B‐CTRL_{\left(n\right)}+a\;*\;H2B‐CTRL_{\left(sn\right)}=H2B‐CTRL_{\left(t\right)}$$

$$H2B‐Dox_{\left(n\right)}+a\;*\;H2B‐Dox_{\left(sn\right)}=H2B‐Dox_{\left(t\right)},$$

to yield *a*:

$$a=\left[H2A‐CTRL_{\left(n\right)}‐H2A‐Dox_{\left(n\right)}\right]/\left[H2A‐Dox_{\left(sn\right)}‐H2A‐CTRL_{\left(sn\right)}\right],$$

where *a* is a constant used to convert protein amount measured by MS to fluorescence intensity values measured by LSC. In the equations, *sn*, *n* and *t* designate *supernatant*, *nuclear* and *total*, respectively. S8 Fig was constructed by plotting the amounts of H2A and H2B detected in the supernatant and that remaining in the nuclei, in %.

### Drug treatments

Doxorubicin, puromycin, cycloheximide, leptomycin B, PYR-41, α-amanitin, actinomycin D, 2-D08 (all Sigma, Budapest, Hungary), 17-AAG (Reagents Direct, Encinitas, USA), MLN4924 (EMD Millipore, Darmstadt, Germany) and MLN7243 (Chemgood, Glen Allen, USA) were diluted to the final concentrations indicated in the Figures and added to live cells in complete DMEM medium for the time indicated, prior to fixing and lysis.

### Preparation of nuclei

The agarose-embedded cells at the bottom of the wells were washed with 500 μl ice cold 1 x PBS, three times for three minutes, then lysis/permeabilization was carried out: (I) Samples were pre-fixed with 400 μl ice cold 4% (m/m) formaldehyde dissolved in 1 x PBS/EDTA on ice for 15 minutes and subsequently permeabilized by replacing the fixative with 500 μl ice cold 1% (v/v) Triton X-100 dissolved in 1 x PBS/EDTA (5 mM EDTA in PBS), for 10 minutes; (II) Samples were lyzed with ice cold 1% (v/v) Triton X-100 dissolved in 1 x PBS/EDTA; (III) Samples were lyzed with 500 μl ice cold 1% (v/v) Triton X-100 dissolved in 300 mM sucrose, 5 mM EDTA. The lysis/permeabilization step was repeated once more, then nuclei were washed with 500 μl ice cold 1 x PBS/EDTA 3x, for three min each.

### Immunofluorescence labeling

After lysis/permeabilization the samples were incubated with 500 μl 5% (m/v) Blotto Non-Fat Dry Milk (Santa Cruz Biotechnology Inc., Santa Cruz, California, USA) in 1 x PBS/EDTA for 30 minutes on ice, to decrease non-specific binding of the antibodies. The blocking solution was washed out with 500 μl ice cold 1 x PBS/EDTA three times for three minutes and indirect immunofluorescence labeling was performed using mouse monoclonal anti-H2B (ab52484, Abcam, Cambridge, UK; 1 mg/ml), rabbit polyclonal anti-H2B (Sigma-Aldrich; 1 mg/ml) or rabbit polyclonal anti-H2A (ab18255, Abcam, Cambridge, UK; 1 mg/ml). Primary antibodies, all diluted in 150 μl of 1 x PBS/EDTA/1% BSA (1 x PBS/ EDTA supplemented with 1% w/v bovine serum albumin), at 4°C, overnight. All the above antibodies were applied to the wells at a titer of 1:800. After labeling with the primary antibodies, the nuclei were washed with 500 μl ice cold 1 x PBS/EDTA three times for 10 minutes. Labeling with the secondary antibodies was performed in 150 μl 1 x PBS/EDTA for 2 hrs on ice, using Alexa fluor 488 (A488) or Alexa fluor 647 (A647) conjugated goat anti-mouse IgG or goat anti-rabbit IgG antibodies (Thermo Fisher Scientific, Waltham, Massachusetts, USA), with identical results. In the figures shown H2A was detected by A647 (red channel) and H2B by A488 (green channel). The secondary antibodies were also used at a titer of 1:800, diluted in 1 x PBS/EDTA from 2 mg/ml stock solutions. After labeling with the secondary antibodies, the agarose-embedded nuclei were washed with 500 μl ice cold 1 x PBS/EDTA three times for 10 minutes. Then the samples were stained with 200 μl 12.5 μg/ml PI (dissolved in 1 x PBS/EDTA) for 60 minutes, on ice. The stained nuclei were washed 3 times with 500 μl ice cold 1 x PBS/EDTA for 3 minutes. Fluorescence intensity distributions were recorded using an iCys LSC, as described below.

### Confocal microscopy

Imaging was carried out with an Olympus FluoView 1000 CLSM equipped with 488 and 633 nm lasers, using a 60x oil immersion oil objective. Composite images were constructed and evaluated using ImageJ software. Instrument settings (laser power, photomultiplier tube voltage, gains, pixel dwell time) and image analyses parameters (brightness, contrast, gamma factor of ImageJ) were identical in the case of all the samples compared in a particular experiment. In S1 Fig, the PI signal was overamplified, as stated there, to demonstrate the separation of histones and DNA upon Dox treatment. Nuclear H2B content was calculated by selecting the nuclei on stack images and determining their total H2B fluorescence integral. Cytoplasmic H2B was calculated by subtracting nuclear from the total intracellular H2B fluorescence integral (8–10 representative cells /sample).

### Automated microscopy (LSC)

Automated microscopic imaging was performed using an iCys instrument (iCys® Research Imaging Cytometer; CompuCyte, Westwood, Massachusetts, USA). A488 and PI were excited using a 488 nm Argon ion laser, A647 was excited with a 633 nm HeNe laser. The fluorescence signals were collected via an UPlan FI 20 x (NA 0.5) objective. A488 was detected through 510/21 nm and 530/30 nm filters, respectively, while A647 and PI were detected through a 650/LP nm filter. Each field (comprising 1000 x 768 pixels) was scanned with a step size of 1.5 μm. Data evaluation and hardware control were performed with the iCys 7.0 software for Windows XP. Gating of G_1_ phase nuclei was according to the fluorescence intensity distribution of the DNA labeled with PI. The integral fluorescence intensity values, representing the summed fluorescence intensity of all the pixels representing the nuclei, were measured and averaged by LSC. The symbols represent measured points, while the lines show the best fit calculated as described below.

Analysis of the curves was by SigmaPlot 12.0, using the ‘Sigmoid 3-parameter’ curve-fitting subroutine. Curves in Fig 1C, S5 and S6 Figs were normalized to ‘1’ dividing the mean fluorescence intensities of G_1_ nuclei treated with different concentrations of Dox by that of the non-treated sample before fitting. 200–1000 G_1_ phase nuclei/well, distinguished based on DNA content as well as circularity, were analyzed out of the 500–2000 nuclei scanned per well. All the SEM values indicated in the Figure were calculated from the datapoints of the cell population analyzed in a representative experiment.

### Viability tests and isobologram analysis

Viability was measured immediately after Dox treatment by incubating the samples with 2 μg/ml PI and Annexin V-FITC (MBL, Woburn, England) according to the manufacturer’s instructions.

Delayed cytotoxicity was measured by the resazurin viability assay. Resazurin (from Sigma-Aldrich Hungary) stock solution was prepared by dissolving 1 mg of its sodium salt in 1 ml of sterile PBS and stored at -20°C. Treatment of Jurkat cells in complete medium with Dox and PYR-41 was carried out in 24-well plates. Increasing concentrations of Dox (0–72 μM) or PYR-41 (0–100 μM) alone were used to determine the dose-response curves of the single agents. For combination treatment, a fixed concentration of PYR-41 (50 μM) and a concentration series of Dox were applied. Treatments were performed for 2 hrs, when cytotoxicity was measured based on mitochondrial function using the resazurin based assay [66]. The cells were mixed with resazurin in colorless RPMI-1640 medium to a final concentration of 18 μM of resazurin and 20,000 cells per well of the 96-well flat-bottom microplates. The plates were incubated at 37°C for 24 h and the fluorescence signals were measured at 530–560/590 nm using a microplate reader (Synergy H1, BioTek). Viability was expressed as the fraction of fluorescence in the treated samples, as compared to the control. Affected fraction, the input parameter of isobologram analyses, was calculated by subtracting the viability values from one. Isobologram analysis was used as a method for identifying the combined effect of multiple drugs in terms of additive, synergistic, or antagonistic effects. IC_50_ (the dose of drug causing 50% cytotoxicity) values were determined and a normalized isobologram was created for the two drugs at their IC_50_ using CompuSyn ver. 1.0 (ComboSyn Inc., Paramus, NJ, USA). If the combination data points fall on the hypothenuse, an additive effect is indicated. If the combination data points fall on the lower left or on the upper right side, synergism or antagonism is indicated, respectively.

### Cell proliferation assay

The CellVue^TM^ NIR780 Cell Labeling Kit was purchased from Thermo Scientific (Waltham, MA, USA). The dye-dilution cell proliferation assay was carried out according to the manufacturer’s directions. Briefly, Jurkat cells were washed once with serum-free medium and re-suspended in Diluent C at 1.8 x 10^7^ cells/ml and the dye was added at 6 μM final concentration. Labeling was stopped by adding 1 ml of serum to each 1 ml sample. After washing with serum containing medium to remove any remaining unbound dye, the samples were treated with 2 and 4 μM Dox or co-treated with 2 μM Dox + 6 μM PYR-41 for 2 hrs. The samples were extensively washed and 2.5 μg/ml PI was added prior to flow-cytometric analyses using a Becton Dickinson FACSAria III Cell Sorter (Becton Dickinson, Mountain View, CA, USA). The CellVue dye was excited using the 633 nm line of a solid state laser and the emitted light was detected using a 780/60 band-pass filter. PI fluorescence was measured by excitation at 531 nm and emission detection using a 660/40 nm band-pass filter. Fluorescence signals were collected in logarithmic mode and the flow-cytometric data were analyzed by the Flowing Software (2.5.1 version).

## Supporting information

S1 File (ZIP)

S1 Fig (A) Single-channel confocal microscopic images of Fig 1E. (B) B: Cell treated with 36 μM Dox, with the PI signal over-amplified.
(TIF)

S2 Fig Single channel confocal microscopic images of Fig 2. (TIF)

S3 Fig Single channel confocal microscopic images of Fig 3. (TIF)

S4 Fig Single channel confocal microscopic images of Fig 4. (TIF)

S5 Fig LSC aggregation assay of H2A. H2A levels after treatment with different concentrations of Dox alone (continuous line) or in the presence of 10 μM Z-VAD-FMK (caspase inhibitor) (dashed line). Fluorescence intensities were normalized to the intensity of untreated samples. Error bars show SEM values.
(TIF)

S6 Fig LSC aggregation assay of H2B. Intranuclear H2B levels after treating live cells with different concentrations of Dox alone (continuous line) and with Dox in the presence of 50 μM PYR-41 (dashed line). Fluorescence intensities were normalized to the intensity of untreated samples. Error bars show SEM values.
(TIF)

S7 Fig Effect of Dox treatment on GFP-tagged and antibody labeled H2B. Representative confocal microscopic images of Dox treated H2B-GFP (green) expressor cells labeled with anti-H2B antibody (red).
(TIF)

S8 Fig Redistribution of H2A and H2B after Dox treatment. Fractions of H2A (panel A) and H2B (panel B) remaining in the nuclei or detected in the supernatant (indicated by green and red colors in the chart, respectively). The cell lysates were prepared without agarose-embedding, the histones were detected by MS in the supernatant and by LSC in the nuclei. The fractions shown in panels A and B were calculated as described in Materials and Methods. Representative microscopic images below show the histones remaining in the nuclei in these experiments.
(TIF)

S9 Fig Additional single channel and composite images of antibody labeled H2B in DCs (see Fig 5). (TIF)

## References

[1] drugs.com/monograph/doxorubicin-hydrochloride.html [Internet]. Drugs.com Doxorubicin Hydrochloride; c2019 [cited 2019 Dec 1]. Available from: http://www.drugs.com/monograph/doxorubicin-hydrochloride.html

[2] Chatterjee K, Zhang J, Honbo N, Karliner JS. Doxorubicin cardiomyopathy. Cardiology. 2010;115(2):155–62. 10.1159/000265166 20016174 PMC2848530

[3] Nitiss JL. Targeting DNA topoisomerase II in cancer chemotherapy. Nature reviews Cancer. 2009;9(5):338–50. 10.1038/nrc2607 19377506 PMC2748742

[4] Studzian K, Wasowska M, Piestrzeniewicz MK, Wilmanska D, Szmigiero L, Oszczapowicz I, et al Inhibition of RNA synthesis in vitro and cell growth by anthracycline antibiotics. Neoplasma. 2001;48(5):412–8. . 11845988

[5] Herman EH, Zhang J, Hasinoff BB, Clark JR Jr., Ferrans VJ. Comparison of the structural changes induced by doxorubicin and mitoxantrone in the heart, kidney and intestine and characterization of the Fe(III)-mitoxantrone complex. Journal of molecular and cellular cardiology. 1997;29(9):2415–30. Epub 1997/09/23. 10.1006/jmcc.1997.0477 . 9299365

[6] Agnieszka Chrustek MI, Hałas Marta, Klimaszewska-Wiśniewska Anna, Gagat Maciej, Grzanka Alina. The influence of doxorubicin on nuclear and cytoplasmic pool of F-actin in the A549 cell line. Medical and Biological Sciences. 2014;28:11–8. 10.12775/MBS.2014.010

[7] Kawaguchi T, Takemura G, Kanamori H, Takeyama T, Watanabe T, Morishita K, et al Prior starvation mitigates acute doxorubicin cardiotoxicity through restoration of autophagy in affected cardiomyocytes. Cardiovascular research. 2012;96(3):456–65. 10.1093/cvr/cvs282 . 22952253

[8] Li DL, Wang ZV, Ding G, Tan W, Luo X, Criollo A, et al Doxorubicin Blocks Cardiomyocyte Autophagic Flux by Inhibiting Lysosome Acidification. Circulation. 2016;133(17):1668–87. 10.1161/CIRCULATIONAHA.115.017443 26984939 PMC4856587

[9] Frederick CA, Williams LD, Ughetto G, van der Marel GA, van Boom JH, Rich A, et al Structural comparison of anticancer drug-DNA complexes: adriamycin and daunomycin. Biochemistry. 1990;29(10):2538–49. . 2334681

[10] Rabbani A, Finn RM, Thambirajah AA, Ausio J. Binding of antitumor antibiotic daunomycin to histones in chromatin and in solution. Biochemistry. 2004;43(51):16497–504. 10.1021/bi048524p . 15610044

[11] Rabbani A, Finn RM, Ausio J. The anthracycline antibiotics: antitumor drugs that alter chromatin structure. BioEssays: news and reviews in molecular, cellular and developmental biology. 2005;27(1):50–6. 10.1002/bies.20160 . 15612030

[12] Scaglioni L, Mondelli R, Artali R, Sirtori FR, Mazzini S. Nemorubicin and doxorubicin bind the G-quadruplex sequences of the human telomeres and of the c-MYC promoter element Pu22. Biochimica et biophysica acta. 2016;1860(6):1129–38. 10.1016/j.bbagen.2016.02.011 . 26922833

[13] Pang B, Qiao X, Janssen L, Velds A, Groothuis T, Kerkhoven R, et al Drug-induced histone eviction from open chromatin contributes to the chemotherapeutic effects of doxorubicin. Nature communications. 2013;4:1908 10.1038/ncomms2921 23715267 PMC3674280

[14] Yang F, Teves SS, Kemp CJ, Henikoff S. Doxorubicin, DNA torsion, and chromatin dynamics. Biochimica et biophysica acta. 2014;1845(1):84–9. 10.1016/j.bbcan.2013.12.002 24361676 PMC3927826

[15] Imre L, Simandi Z, Horvath A, Fenyofalvi G, Nanasi P, Niaki EF, et al Nucleosome stability measured in situ by automated quantitative imaging. Scientific reports. 2017;7(1):12734 10.1038/s41598-017-12608-9 28986581 PMC5630628

[16] Salerno D, Brogioli D, Cassina V, Turchi D, Beretta GL, Seruggia D, et al Magnetic tweezers measurements of the nanomechanical properties of DNA in the presence of drugs. Nucleic acids research. 2010;38(20):7089–99. 10.1093/nar/gkq597 20601682 PMC2978368

[17] Almaqwashi AA, Paramanathan T, Rouzina I, Williams MC. Mechanisms of small molecule-DNA interactions probed by single-molecule force spectroscopy. Nucleic acids research. 2016;44(9):3971–88. 10.1093/nar/gkw237 27085806 PMC4872107

[18] Chaires JB, Dattagupta N, Crothers DM. Studies on interaction of anthracycline antibiotics and deoxyribonucleic acid: equilibrium binding studies on interaction of daunomycin with deoxyribonucleic acid. Biochemistry. 1982;21(17):3933–40. 10.1021/bi00260a005 . 7126524

[19] Yang F, Kemp CJ, Henikoff S. Anthracyclines induce double-strand DNA breaks at active gene promoters. Mutation research. 2015;773:9–15. 10.1016/j.mrfmmm.2015.01.007 25705119 PMC4332850

[20] Wojcik K, Zarebski M, Cossarizza A, Dobrucki JW. Daunomycin, an antitumor DNA intercalator, influences histone-DNA interactions. Cancer biology & therapy. 2013;14(9):823–32. 10.4161/cbt.25328 23792590 PMC3909551

[21] Sprigg L, Li A, Choy FY, Ausio J. Interaction of daunomycin with acetylated chromatin. Journal of medicinal chemistry. 2010;53(17):6457–65. 10.1021/jm1007853 . 20698509

[22] Wojcik K, Dobrucki JW. Interaction of a DNA intercalator DRAQ5, and a minor groove binder SYTO17, with chromatin in live cells—influence on chromatin organization and histone-DNA interactions. Cytometry Part A: the journal of the International Society for Analytical Cytology. 2008;73(6):555–62. 10.1002/cyto.a.20573 . 18459157

[23] Rabbani A, Iskandar M, Ausio J. Daunomycin-induced unfolding and aggregation of chromatin. The Journal of biological chemistry. 1999;274(26):18401–6. 10.1074/jbc.274.26.18401 . 10373446

[24] Hsu LW, Chen CL, Nakano T, Lai CY, Chiang KC, Lin YC, et al The role of a nuclear protein, histone H1, on signalling pathways for the maturation of dendritic cells. Clinical and experimental immunology. 2008;152(3):576–84. Epub 2008/04/26. 10.1111/j.1365-2249.2008.03652.x 18435805 PMC2453206

[25] Rozijn TH, Tonino GJ. STUDIES ON THE YEAST NUCLEUS. I. THE ISOLATION OF NUCLEI. Biochimica et biophysica acta. 1964;91:105–12. Epub 1964/09/11. 10.1016/0926-6550(64)90174-4 . 14227256

[26] Ullrich O, Grune T. Proteasomal degradation of oxidatively damaged endogenous histones in K562 human leukemic cells. Free radical biology & medicine. 2001;31(7):887–93. Epub 2001/10/05. 10.1016/s0891-5849(01)00672-4 . 11585707

[27] Yang Y, Kitagaki J, Dai RM, Tsai YC, Lorick KL, Ludwig RL, et al Inhibitors of ubiquitin-activating enzyme (E1), a new class of potential cancer therapeutics. Cancer research. 2007;67(19):9472–81. 10.1158/0008-5472.CAN-07-0568 . 17909057

[28] Kamiyama D, Sekine S, Barsi-Rhyne B, Hu J, Chen B, Gilbert LA, et al Versatile protein tagging in cells with split fluorescent protein. Nature communications. 2016;7:11046 Epub 2016/03/19. 10.1038/ncomms11046 26988139 PMC4802074

[29] Matilainen O, Sleiman MSB, Quiros PM. The chromatin remodeling factor ISW-1 integrates organismal responses against nuclear and mitochondrial stress. 2017;8(1):1818 10.1038/s41467-017-01903-8 . 29180639 PMC5703887

[30] Travers J, Rochman M. Chromatin regulates IL-33 release and extracellular cytokine activity. 2018;9(1):3244 10.1038/s41467-018-05485-x . 30108214 PMC6092330

[31] Kobiyama K, Takeshita F, Jounai N, Sakaue-Sawano A, Miyawaki A, Ishii KJ, et al Extrachromosomal histone H2B mediates innate antiviral immune responses induced by intracellular double-stranded DNA. Journal of virology. 2010;84(2):822–32. Epub 2009/11/13. 10.1128/JVI.01339-09 19906922 PMC2798368

[32] Kobiyama K, Kawashima A, Jounai N, Takeshita F, Ishii KJ, Ito T, et al Role of Extrachromosomal Histone H2B on Recognition of DNA Viruses and Cell Damage. Frontiers in genetics. 2013;4:91 Epub 2013/06/05. 10.3389/fgene.2013.00091 23734163 PMC3661947

[33] Iqbal J, Ansari MA, Kumar B, Dutta D, Roy A, Chikoti L, et al Histone H2B-IFI16 Recognition of Nuclear Herpesviral Genome Induces Cytoplasmic Interferon-beta Responses. 2016;12(10):e1005967 10.1371/journal.ppat.1005967 . 27764250 PMC5072618

[34] Szatmary P, Huang W, Criddle D, Tepikin A, Sutton R. Biology, role and therapeutic potential of circulating histones in acute inflammatory disorders. Journal of cellular and molecular medicine. 2018;22(10):4617–29. Epub 2018/08/08. 10.1111/jcmm.13797 30085397 PMC6156248

[35] Jiang L, Smith JN, Anderson SL, Ma P, Mizzen CA, Kelleher NL. Global assessment of combinatorial post-translational modification of core histones in yeast using contemporary mass spectrometry. LYS4 trimethylation correlates with degree of acetylation on the same H3 tail. The Journal of biological chemistry. 2007;282(38):27923–34. 10.1074/jbc.M704194200 . 17652096

[36] Foster ER, Downs JA. Methylation of H3 K4 and K79 is not strictly dependent on H2B K123 ubiquitylation. The Journal of cell biology. 2009;184(5):631–8. Epub 2009/03/04. 10.1083/jcb.200812088 19255247 PMC2686411

[37] Kapuria V, Peterson LF, Showalter HD, Kirchhoff PD, Talpaz M, Donato NJ. Protein cross-linking as a novel mechanism of action of a ubiquitin-activating enzyme inhibitor with anti-tumor activity. Biochemical pharmacology. 2011;82(4):341–9. 10.1016/j.bcp.2011.05.012 . 21621524

[38] Kudo N, Matsumori N, Taoka H, Fujiwara D, Schreiner EP, Wolff B, et al Leptomycin B inactivates CRM1/exportin 1 by covalent modification at a cysteine residue in the central conserved region. Proceedings of the National Academy of Sciences of the United States of America. 1999;96(16):9112–7. 10.1073/pnas.96.16.9112 10430904 PMC17741

[39] Cole CN, Scarcelli JJ. Transport of messenger RNA from the nucleus to the cytoplasm. Current opinion in cell biology. 2006;18(3):299–306. 10.1016/j.ceb.2006.04.006 . 16682182

[40] Moore MJ, Rosbash M. Cell biology. TAPping into mRNA export. Science. 2001;294(5548):1841–2. 10.1126/science.1067676 . 11729289

[41] Muller-McNicoll M, Botti V, de Jesus Domingues AM, Brandl H, Schwich OD, Steiner MC, et al SR proteins are NXF1 adaptors that link alternative RNA processing to mRNA export. Genes & development. 2016;30(5):553–66. 10.1101/gad.276477.115 26944680 PMC4782049

[42] Thomas M, Lischka P, Muller R, Stamminger T. The cellular DExD/H-box RNA-helicases UAP56 and URH49 exhibit a CRM1-independent nucleocytoplasmic shuttling activity. PloS one. 2011;6(7):e22671 10.1371/journal.pone.0022671 21799930 PMC3142171

[43] Zhang H, Mahadevan K, Palazzo AF. Sumoylation is Required for the Cytoplasmic Accumulation of a Subset of mRNAs. Genes. 2014;5(4):982–1000. 10.3390/genes5040982 25333844 PMC4276922

[44] Ashikari D, Takayama K, Tanaka T, Suzuki Y, Obinata D, Fujimura T, et al Androgen induces G3BP2 and SUMO-mediated p53 nuclear export in prostate cancer. Oncogene. 2017;36(45):6272–81. 10.1038/onc.2017.225 . 28692047

[45] da Silva MG, Mattos E, Camacho-Pereira J, Domitrovic T, Galina A, Costa MW, et al Cardiac systolic dysfunction in doxorubicin-challenged rats is associated with upregulation of MuRF2 and MuRF3 E3 ligases. Experimental and clinical cardiology. 2012;17(3):101–9. 23620696 PMC3628421

[46] Fang S, Qiu J, Wu Z, Bai T, Guo W. Down-regulation of UBC9 increases the sensitivity of hepatocellular carcinoma to doxorubicin. Oncotarget. 2017;8(30):49783–95. 10.18632/oncotarget.17939 28572537 PMC5564807

[47] Huang RY, Kowalski D, Minderman H, Gandhi N, Johnson ES. Small ubiquitin-related modifier pathway is a major determinant of doxorubicin cytotoxicity in Saccharomyces cerevisiae. Cancer research. 2007;67(2):765–72. 10.1158/0008-5472.CAN-06-2839 . 17234788

[48] Braunstein M, Liao L, Lyttle N, Lobo N, Taylor KJ, Krzyzanowski PM, et al Downregulation of histone H2A and H2B pathways is associated with anthracycline sensitivity in breast cancer. Breast cancer research: BCR. 2016;18(1):16 10.1186/s13058-016-0676-6 26852132 PMC4744406

[49] Watson K, Edwards RJ, Shaunak S, Parmelee DC, Sarraf C, Gooderham NJ, et al Extra-nuclear location of histones in activated human peripheral blood lymphocytes and cultured T-cells. Biochemical pharmacology. 1995;50(3):299–309. 10.1016/0006-2952(95)00142-m . 7646532

[50] Kim HS, Cho JH, Park HW, Yoon H, Kim MS, Kim SC. Endotoxin-neutralizing antimicrobial proteins of the human placenta. Journal of immunology. 2002;168(5):2356–64. 10.4049/jimmunol.168.5.2356 . 11859126

[51] Xu J, Zhang X, Pelayo R, Monestier M, Ammollo CT, Semeraro F, et al Extracellular histones are major mediators of death in sepsis. Nature medicine. 2009;15(11):1318–21. 10.1038/nm.2053 19855397 PMC2783754

[52] Bleher R, Martin R. Nucleo-cytoplasmic translocation of histone H1 during the HeLa cell cycle. Chromosoma. 1999;108(5):308–16. Epub 1999/10/20. 10.1007/s004120050382 . 10525967

[53] Kawashima A, Tanigawa K, Akama T, Wu H, Sue M, Yoshihara A, et al Fragments of genomic DNA released by injured cells activate innate immunity and suppress endocrine function in the thyroid. Endocrinology. 2011;152(4):1702–12. Epub 2011/02/10. 10.1210/en.2010-1132 . 21303947

[54] Kelly B, O'Neill LA. Metabolic reprogramming in macrophages and dendritic cells in innate immunity. Cell research. 2015;25(7):771–84. Epub 2015/06/06. 10.1038/cr.2015.68 26045163 PMC4493277

[55] Steinman RM, Hemmi H. Dendritic cells: translating innate to adaptive immunity. Current topics in microbiology and immunology. 2006;311:17–58. Epub 2006/10/20. 10.1007/3-540-32636-7_2 . 17048704

[56] Matsuo S, Sharma A, Wang P, Yang WL. PYR-41, A Ubiquitin-Activating Enzyme E1 Inhibitor, Attenuates Lung Injury in Sepsis. Shock. 2018;49(4):442–50. 10.1097/SHK.0000000000000931 28661933 PMC5745315

[57] Barpe DR, Rosa DD, Froehlich PE. Pharmacokinetic evaluation of doxorubicin plasma levels in normal and overweight patients with breast cancer and simulation of dose adjustment by different indexes of body mass. European journal of pharmaceutical sciences: official journal of the European Federation for Pharmaceutical Sciences. 2010;41(3–4):458–63. 10.1016/j.ejps.2010.07.015 . 20688160

[58] Eksborg S. Pharmacokinetics of anthracyclines. Acta oncologica. 1989;28(6):873–6. 10.3109/02841868909092323 . 2611038

[59] Greene RF, Collins JM, Jenkins JF, Speyer JL, Myers CE. Plasma pharmacokinetics of adriamycin and adriamycinol: implications for the design of in vitro experiments and treatment protocols. Cancer research. 1983;43(7):3417–21. 6850648. 6850648

[60] Paul C, Liliemark J, Tidefelt U, Gahrton G, Peterson C. Pharmacokinetics of daunorubicin and doxorubicin in plasma and leukemic cells from patients with acute nonlymphoblastic leukemia. Therapeutic drug monitoring. 1989;11(2):140–8. 10.1097/00007691-198903000-00004 . 2718219

[61] Nesher E, Safina A, Aljahdali I, Portwood S, Wang ES, Koman I, et al Role of Chromatin Damage and Chromatin Trapping of FACT in Mediating the Anticancer Cytotoxicity of DNA-Binding Small-Molecule Drugs. Cancer research. 2018;78(6):1431–43. Epub 2018/01/18. 10.1158/0008-5472.CAN-17-2690 29339544 PMC5856628

[62] Miles JS, Sojourner SJ, Jaafar L, Whitmore A, Darling-Reed S, Flores-Rozas H. THE ROLE OF PROTEIN CHAPERONES IN THE SURVIVAL FROM ANTHRACYCLINE-INDUCED OXIDATIVE STRESS IN SACCHAROMYCES CEREVISIAE. International journal of advanced research. 2018;6(3):144–52. Epub 2018/04/17. 29657945 PMC5894877

[63] Rodrigues PG, Miranda-Silva D, Costa SM, Barros C, Hamdani N, Moura C, et al Early myocardial changes induced by doxorubicin in the nonfailing dilated ventricle. 2019;316(3):H459–h75. 10.1152/ajpheart.00401.2018 . 30525890

[64] Cox J, Mann M. MaxQuant enables high peptide identification rates, individualized p.p.b.-range mass accuracies and proteome-wide protein quantification. Nature biotechnology. 2008;26(12):1367–72. Epub 2008/11/26. 10.1038/nbt.1511 . 19029910

[65] Cox J, Hein MY, Luber CA, Paron I, Nagaraj N, Mann M. Accurate proteome-wide label-free quantification by delayed normalization and maximal peptide ratio extraction, termed MaxLFQ. Molecular & cellular proteomics: MCP. 2014;13(9):2513–26. Epub 2014/06/20. 10.1074/mcp.M113.031591 24942700 PMC4159666

[66] O'Brien J, Wilson I, Orton T, Pognan F. Investigation of the Alamar Blue (resazurin) fluorescent dye for the assessment of mammalian cell cytotoxicity. European journal of biochemistry. 2000;267(17):5421–6. 10.1046/j.1432-1327.2000.01606.x . 10951200

