# Supplementary figures and images for: Doxorubicin induces large-scale and differential H2A and H2B redistribution in live cells
Source: PLoS One. 2020 Apr 16;15(4):e0231223. doi: 10.1371/journal.pone.0231223 (PMC7162453; doi:10.1371/journal.pone.0231223)

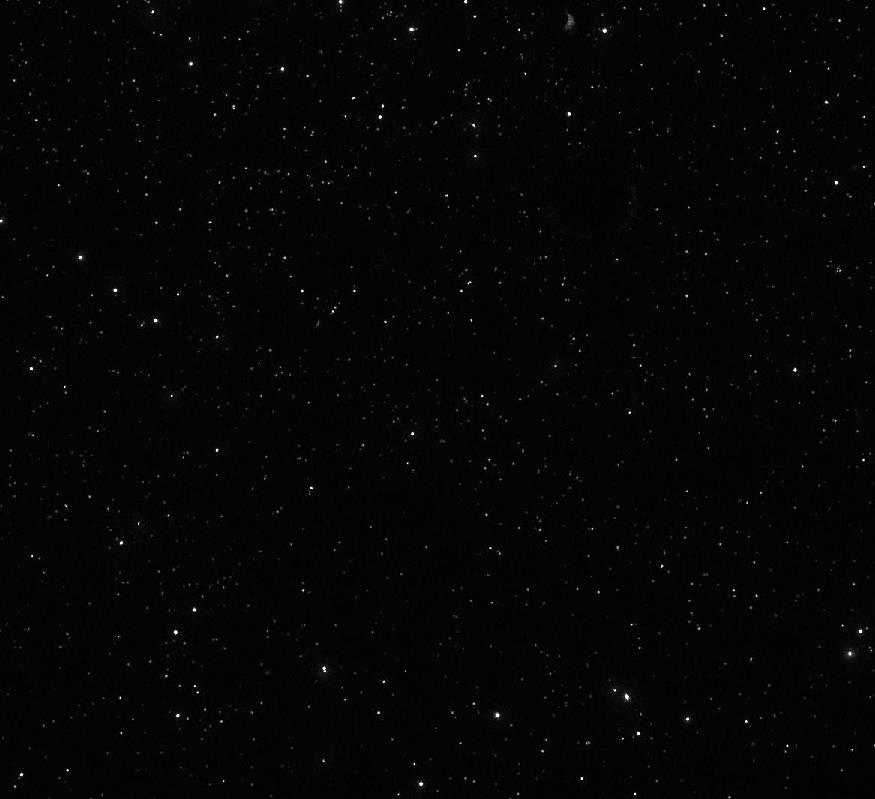

Supplement: S1 File — (ZIP) [file pone.0231223.s001.zip › H2A_Well_Images/H2A_0.5uM_Dox.jpg]

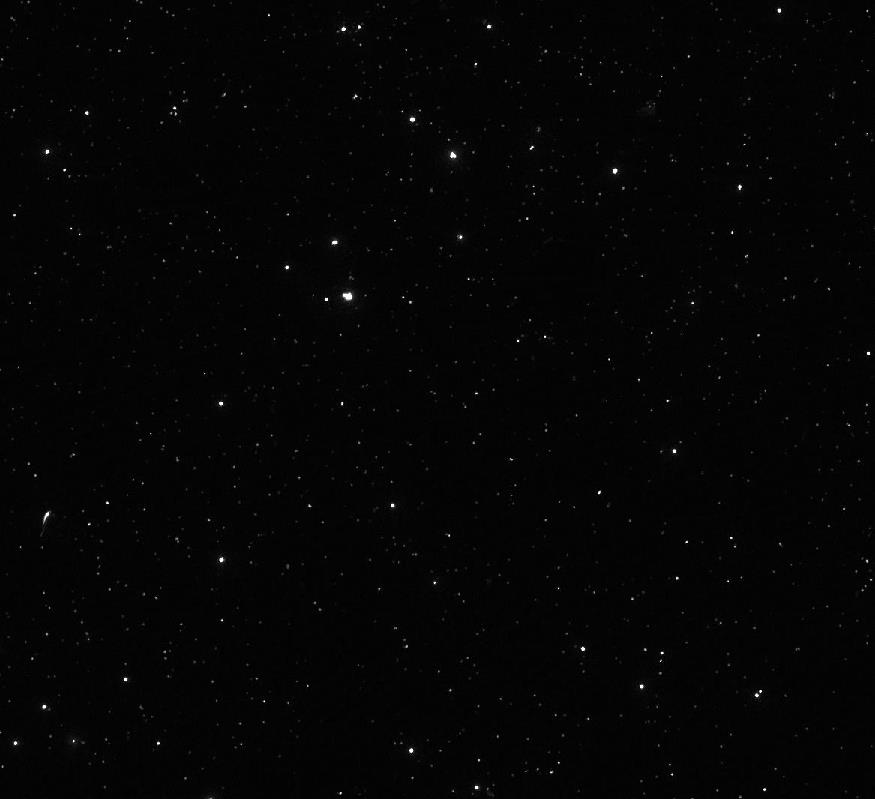

Supplement: S1 File — (ZIP) [file pone.0231223.s001.zip › H2A_Well_Images/H2A_1.13uM_Dox.jpg]

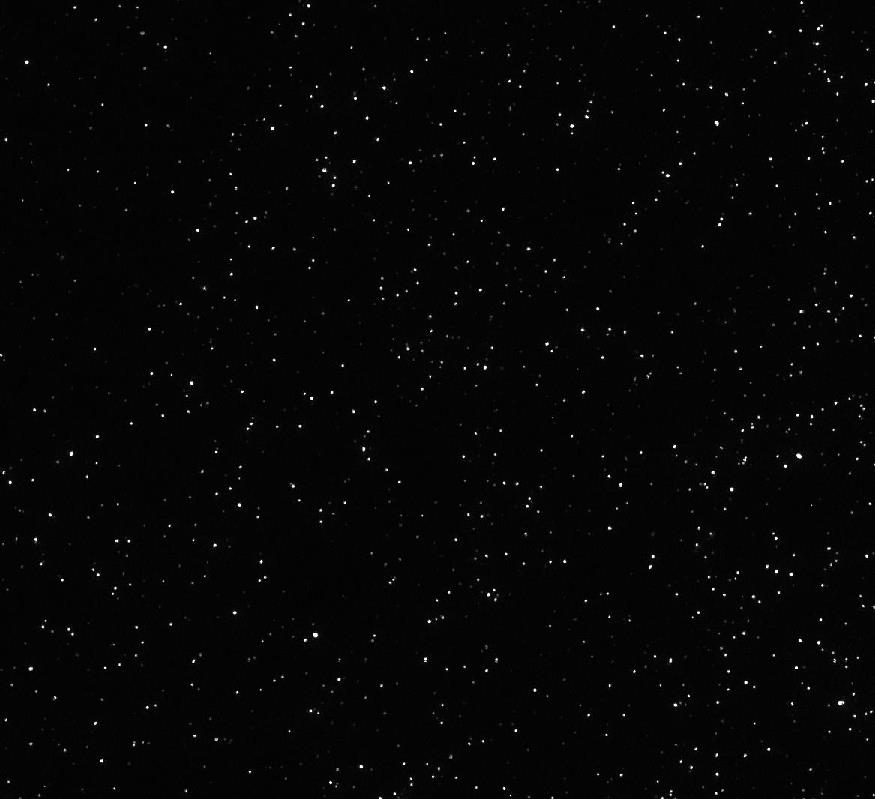

Supplement: S1 File — (ZIP) [file pone.0231223.s001.zip › H2A_Well_Images/H2A_18uM_Dox.jpg]

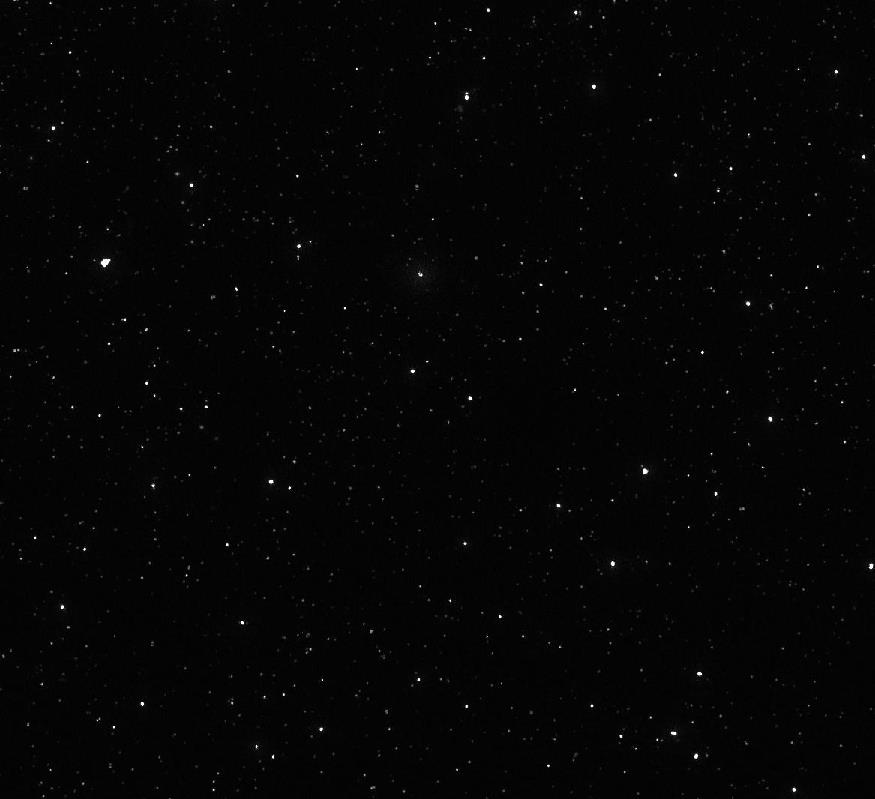

Supplement: S1 File — (ZIP) [file pone.0231223.s001.zip › H2A_Well_Images/H2A_2.25uM_Dox.jpg]

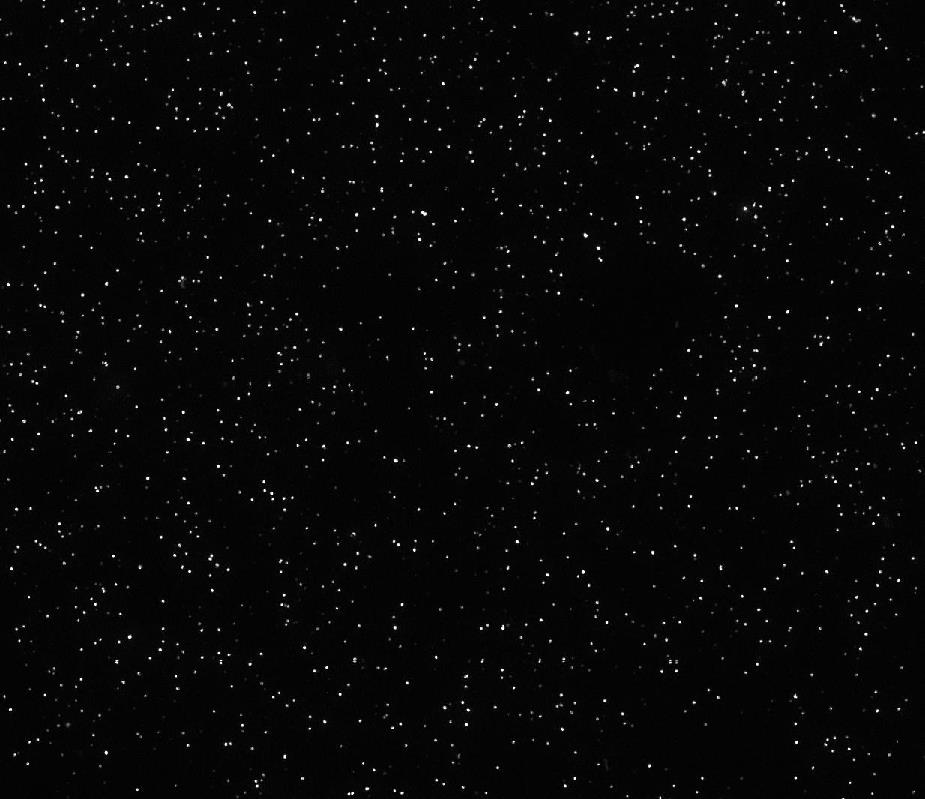

Supplement: S1 File — (ZIP) [file pone.0231223.s001.zip › H2A_Well_Images/H2A_36uM_Dox.jpg]

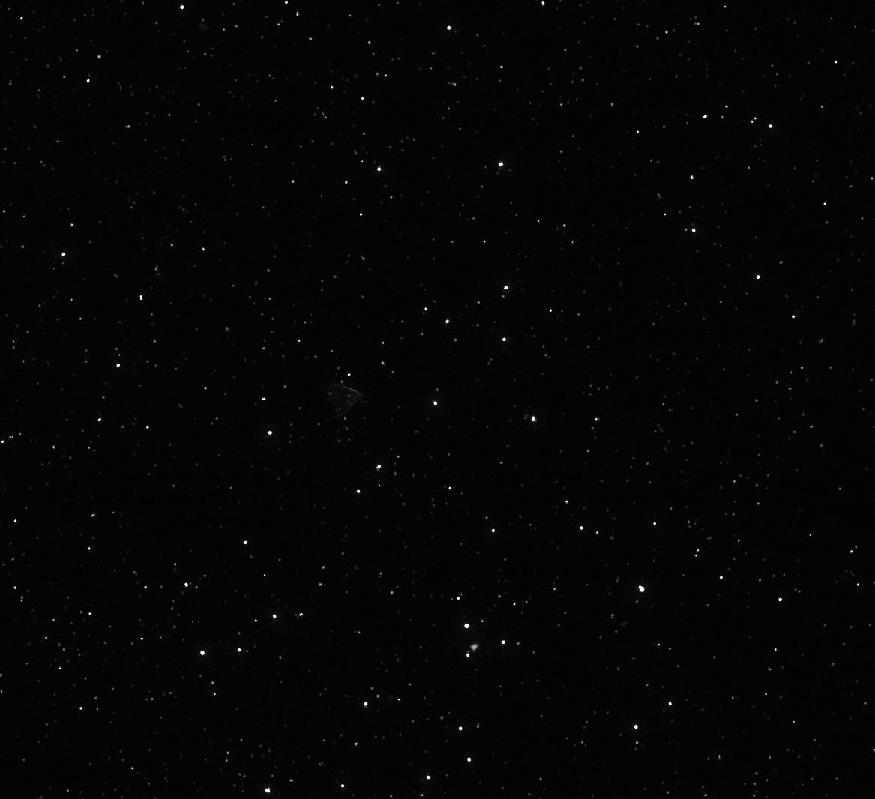

Supplement: S1 File — (ZIP) [file pone.0231223.s001.zip › H2A_Well_Images/H2A_4.5uM_Dox.jpg]

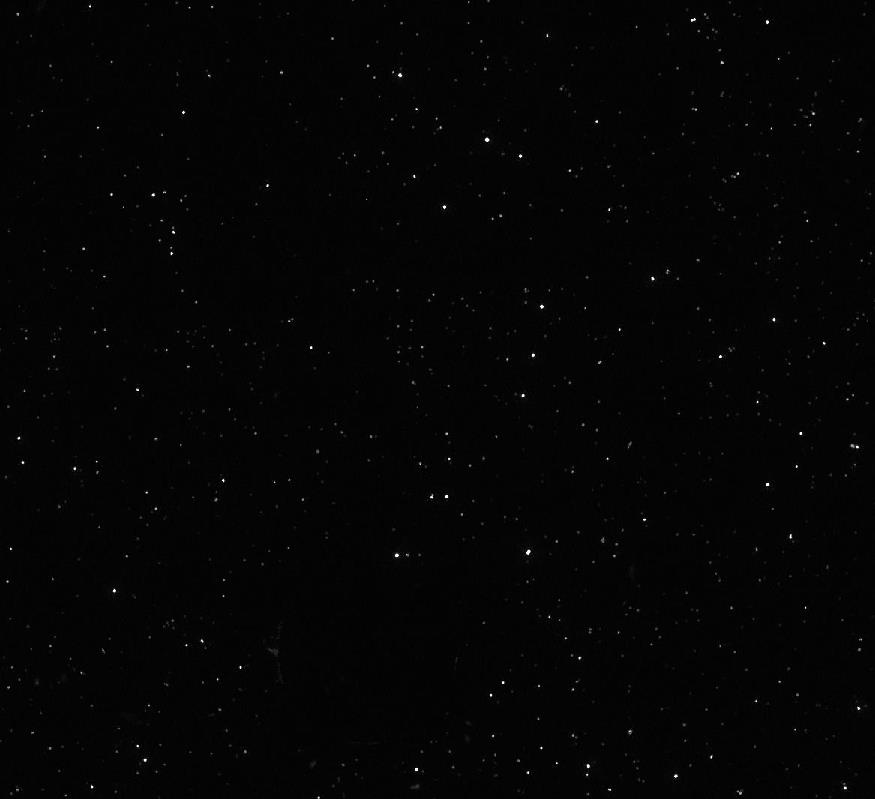

Supplement: S1 File — (ZIP) [file pone.0231223.s001.zip › H2A_Well_Images/H2A_9uM_Dox.jpg]

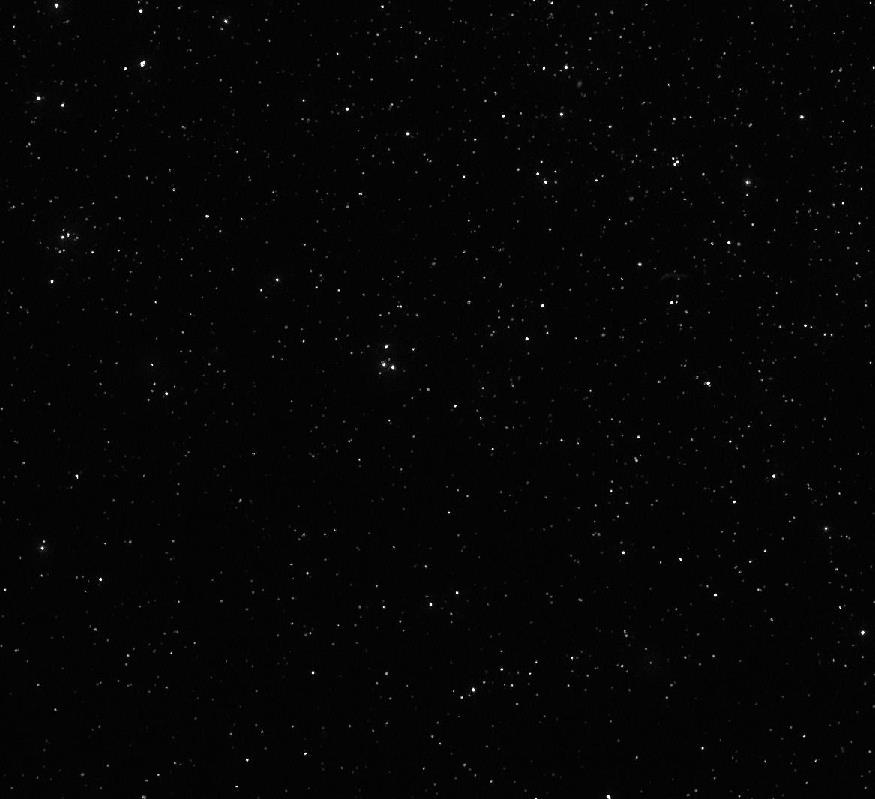

Supplement: S1 File — (ZIP) [file pone.0231223.s001.zip › H2A_Well_Images/H2A_Control.jpg]

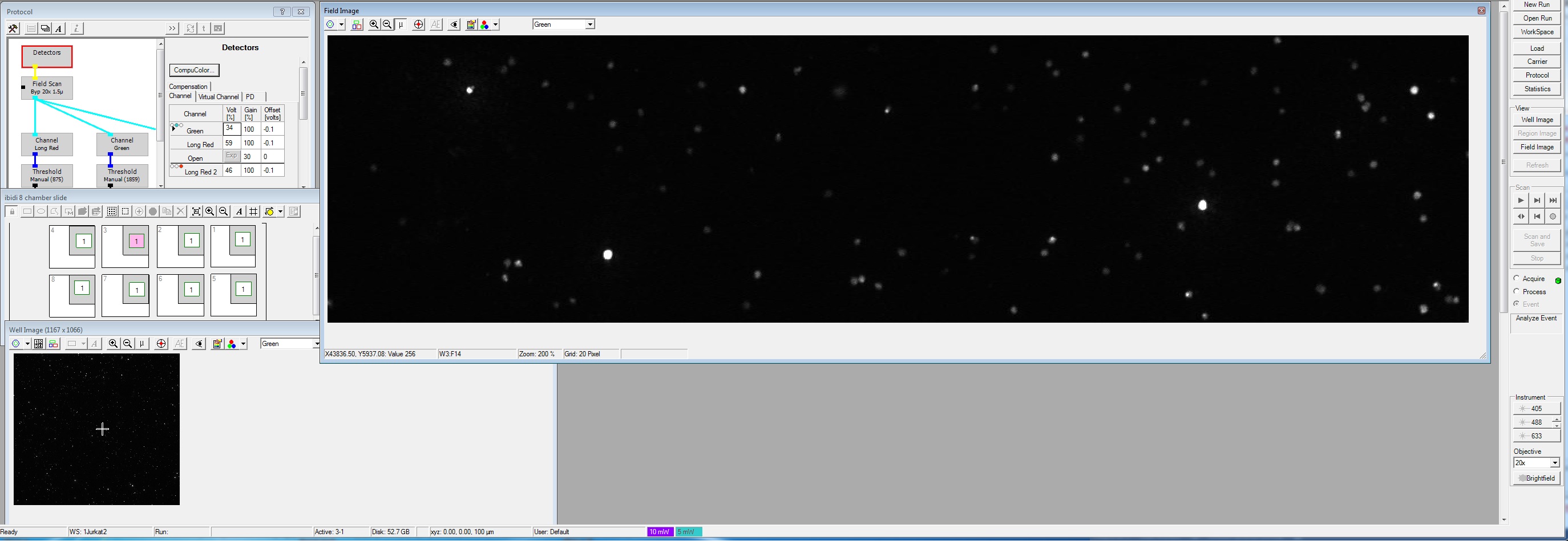

Supplement: S1 File — (ZIP) [file pone.0231223.s001.zip › Representative_H2A_Field_Images/H2A_0.5uM_Dox.jpg]

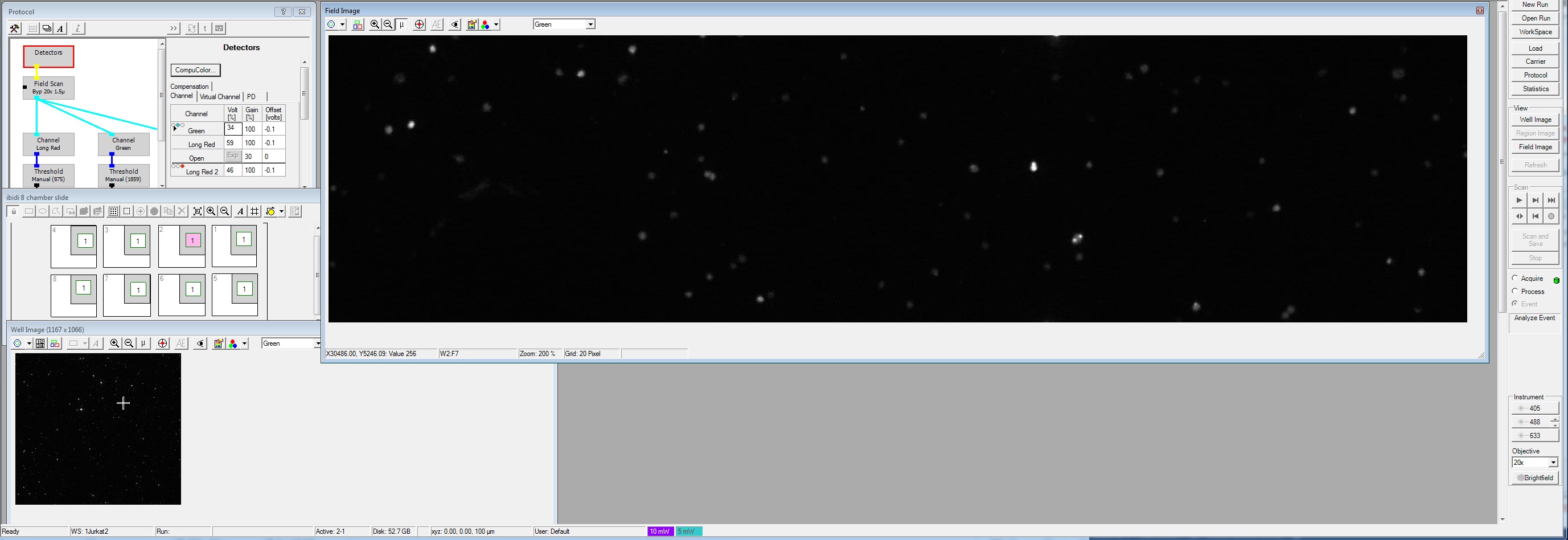

Supplement: S1 File — (ZIP) [file pone.0231223.s001.zip › Representative_H2A_Field_Images/H2A_1.13uM_Dox.jpg]

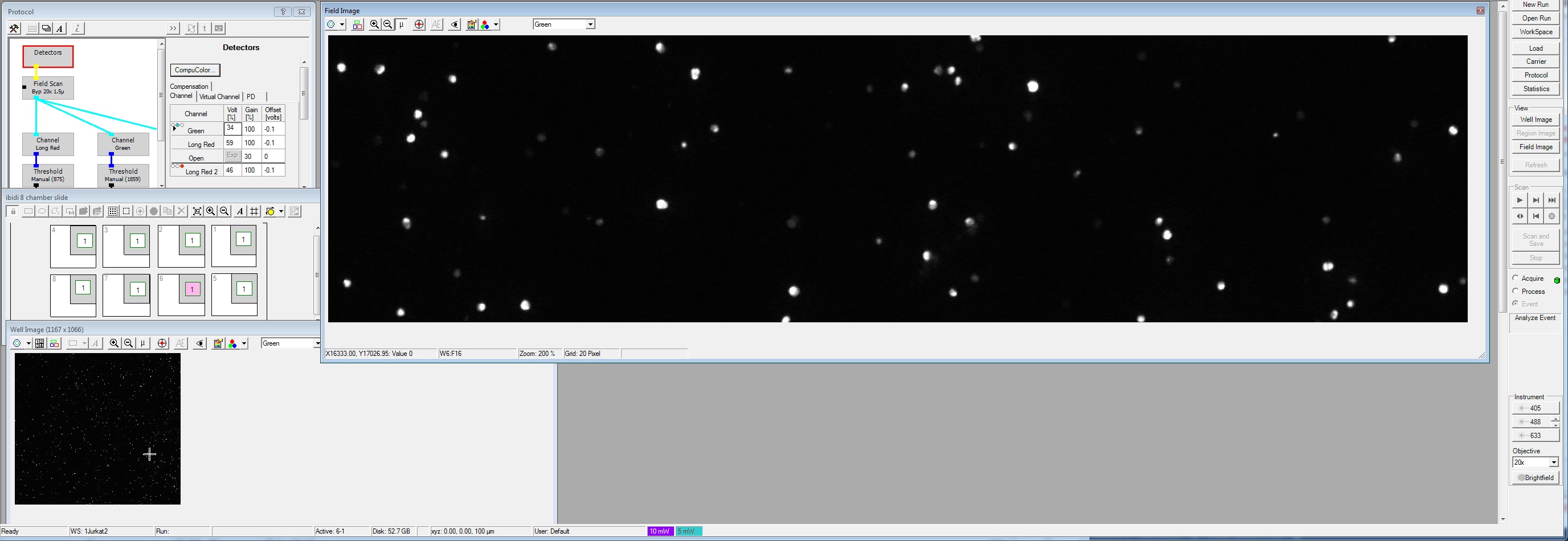

Supplement: S1 File — (ZIP) [file pone.0231223.s001.zip › Representative_H2A_Field_Images/H2A_18uM_Dox.jpg]

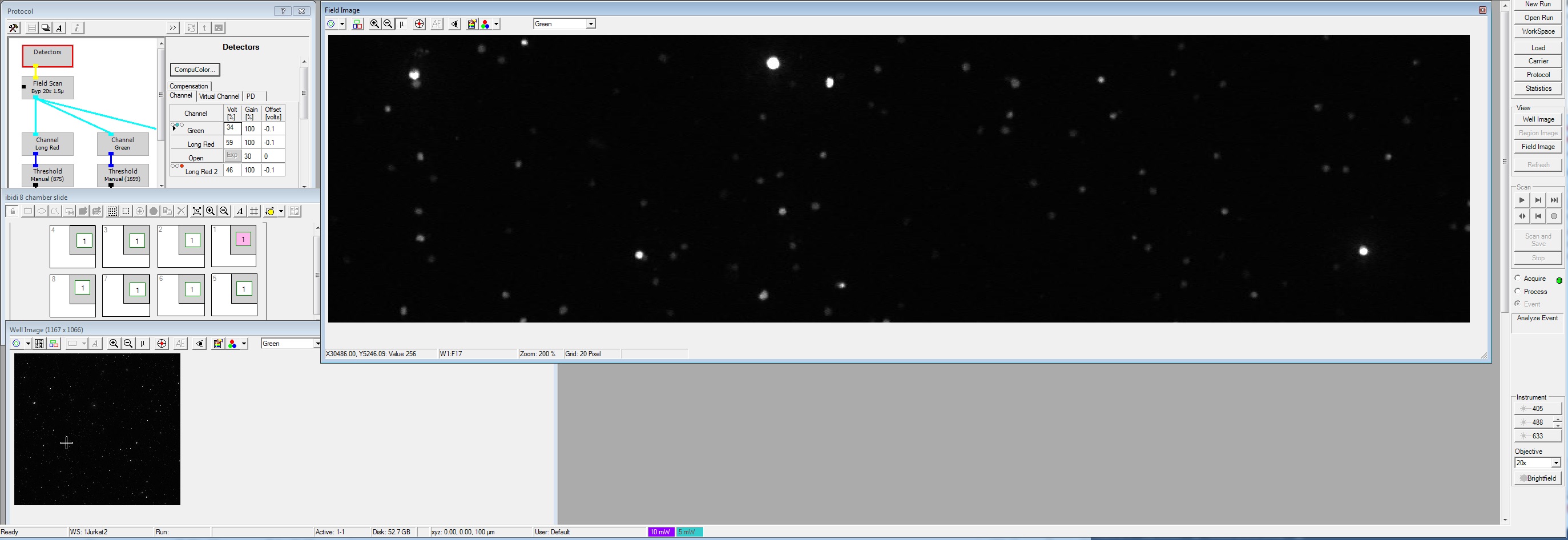

Supplement: S1 File — (ZIP) [file pone.0231223.s001.zip › Representative_H2A_Field_Images/H2A_2.25uM_Dox.jpg]

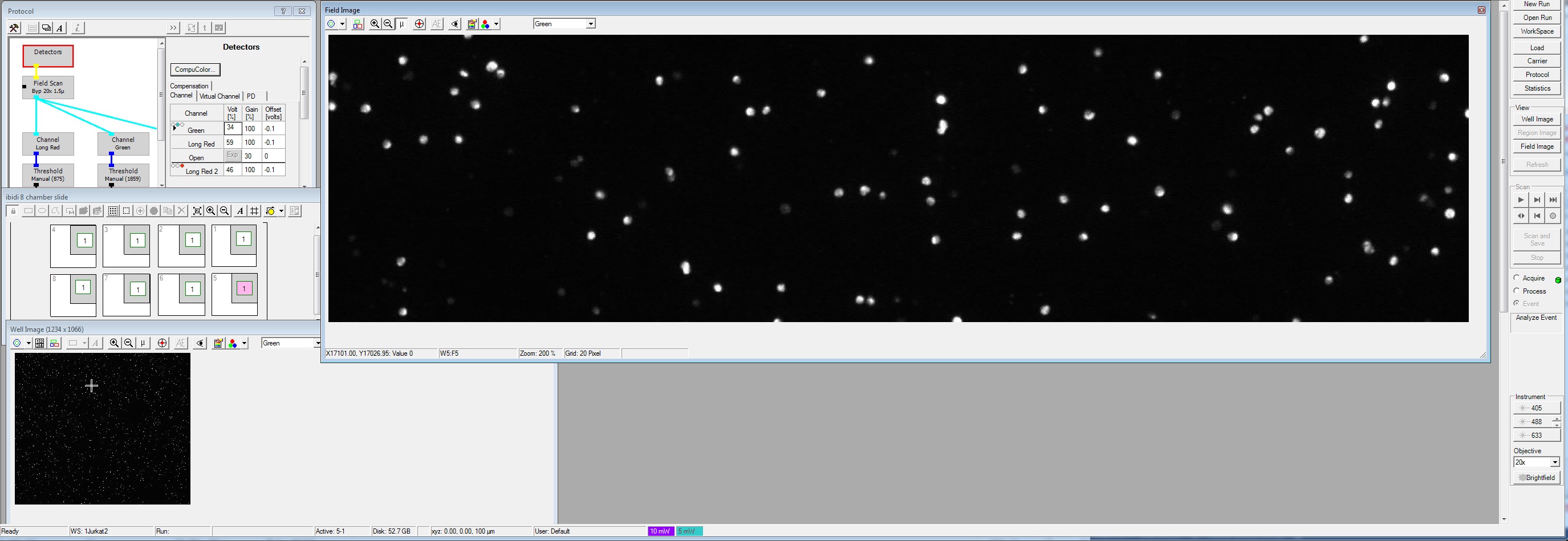

Supplement: S1 File — (ZIP) [file pone.0231223.s001.zip › Representative_H2A_Field_Images/H2A_36uM_Dox.jpg]

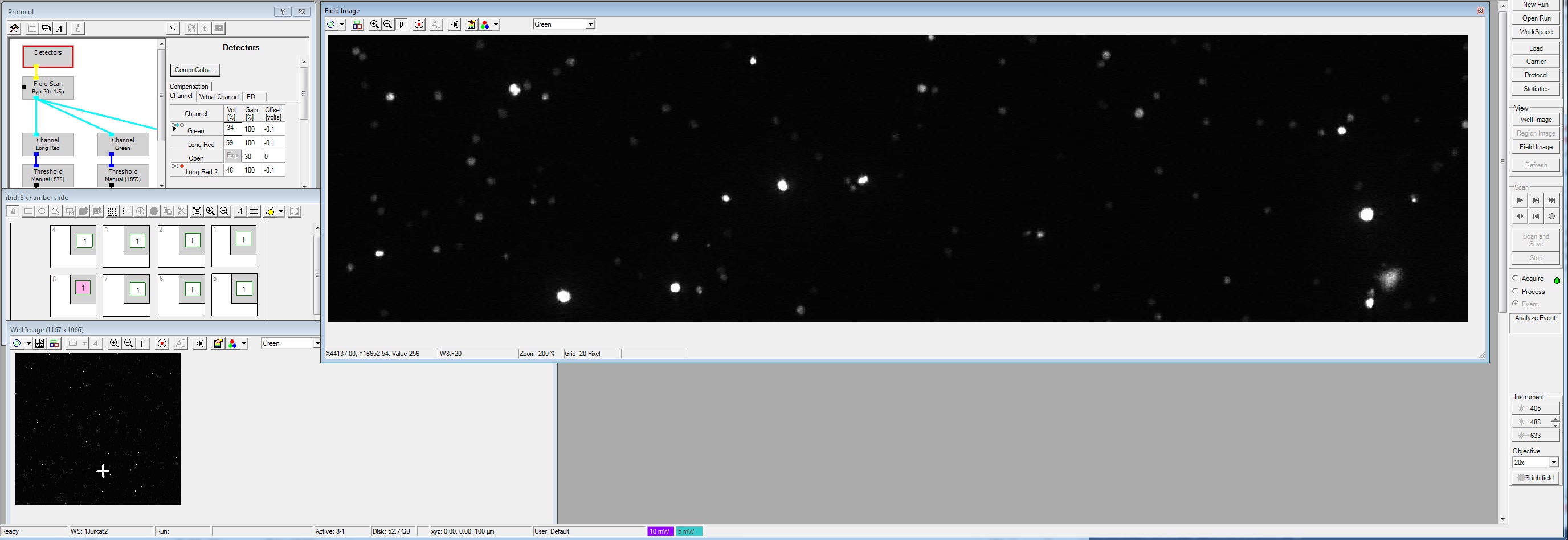

Supplement: S1 File — (ZIP) [file pone.0231223.s001.zip › Representative_H2A_Field_Images/H2A_4.5uM_Dox.jpg]

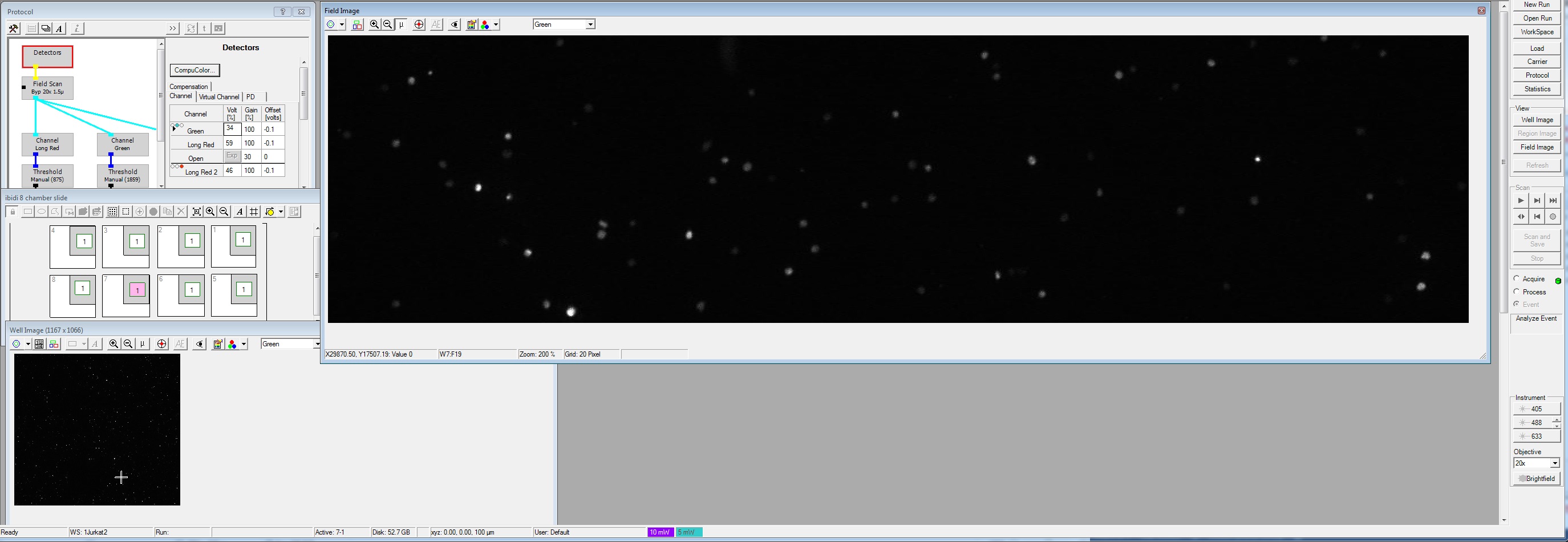

Supplement: S1 File — (ZIP) [file pone.0231223.s001.zip › Representative_H2A_Field_Images/H2A_9uM_Dox.jpg]

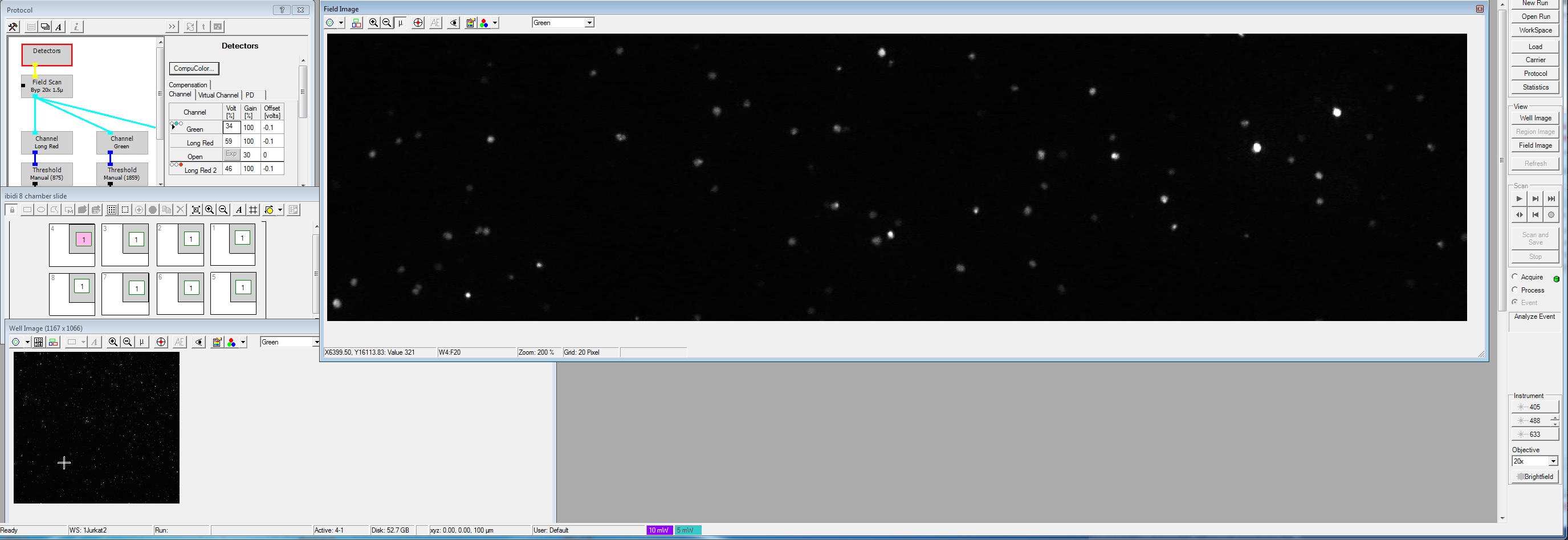

Supplement: S1 File — (ZIP) [file pone.0231223.s001.zip › Representative_H2A_Field_Images/H2A_Control.jpg]

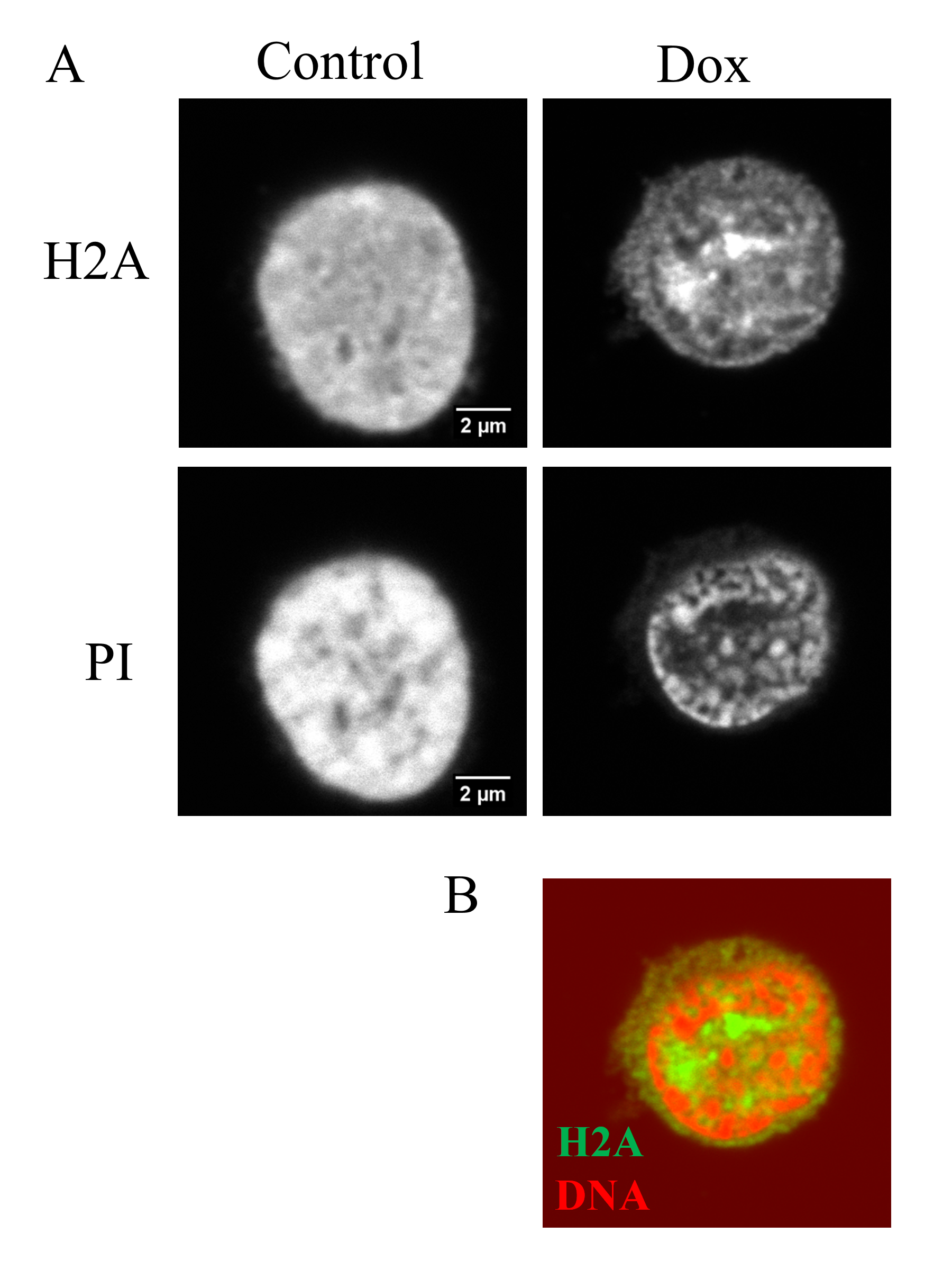

Supplement: S1 Fig — (A) Single-channel confocal microscopic images of Fig 1E. (B) B: Cell treated with 36 μM Dox, with the PI signal over-amplified. (TIF) [file pone.0231223.s002.tif]

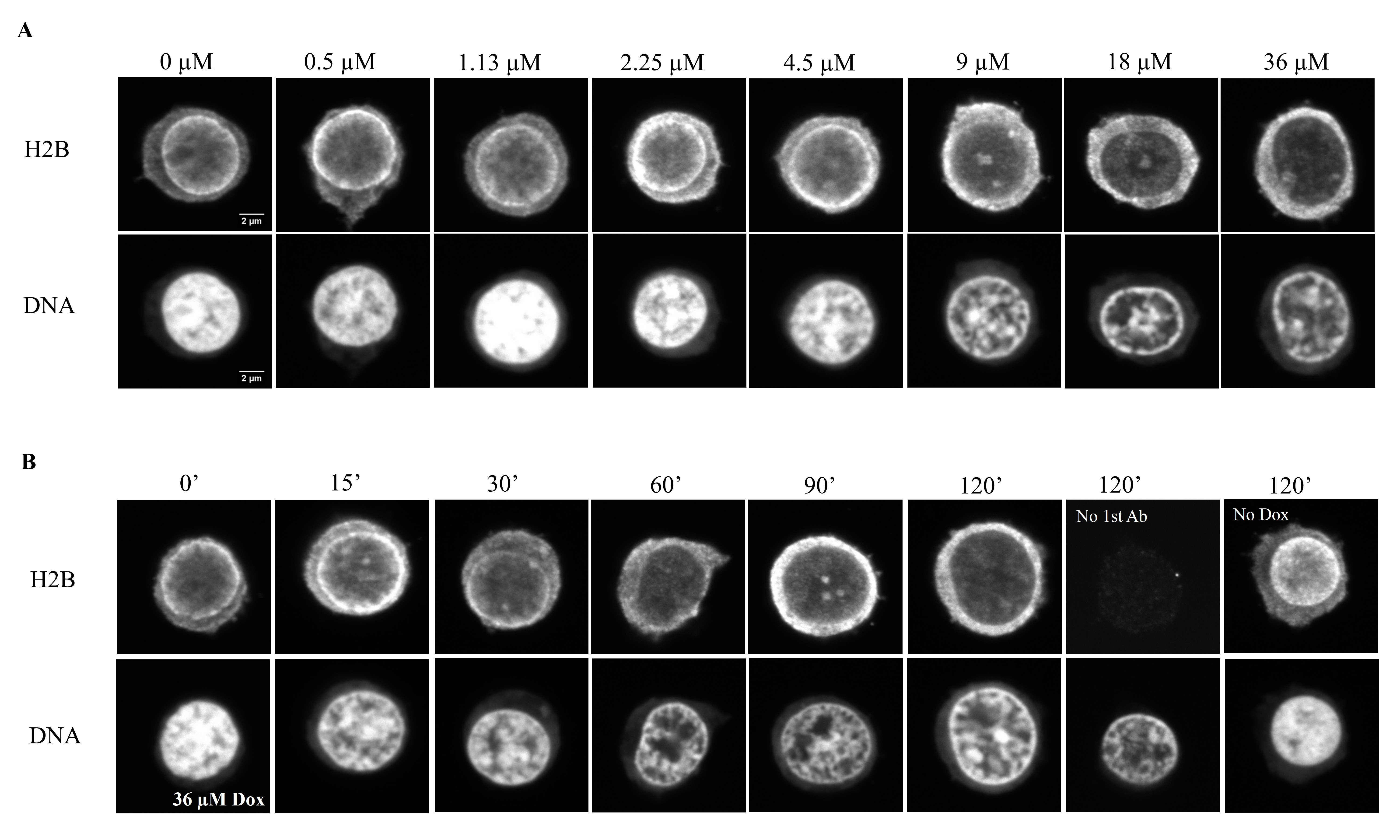

Supplement: S2 Fig — (TIF) [file pone.0231223.s003.tif]

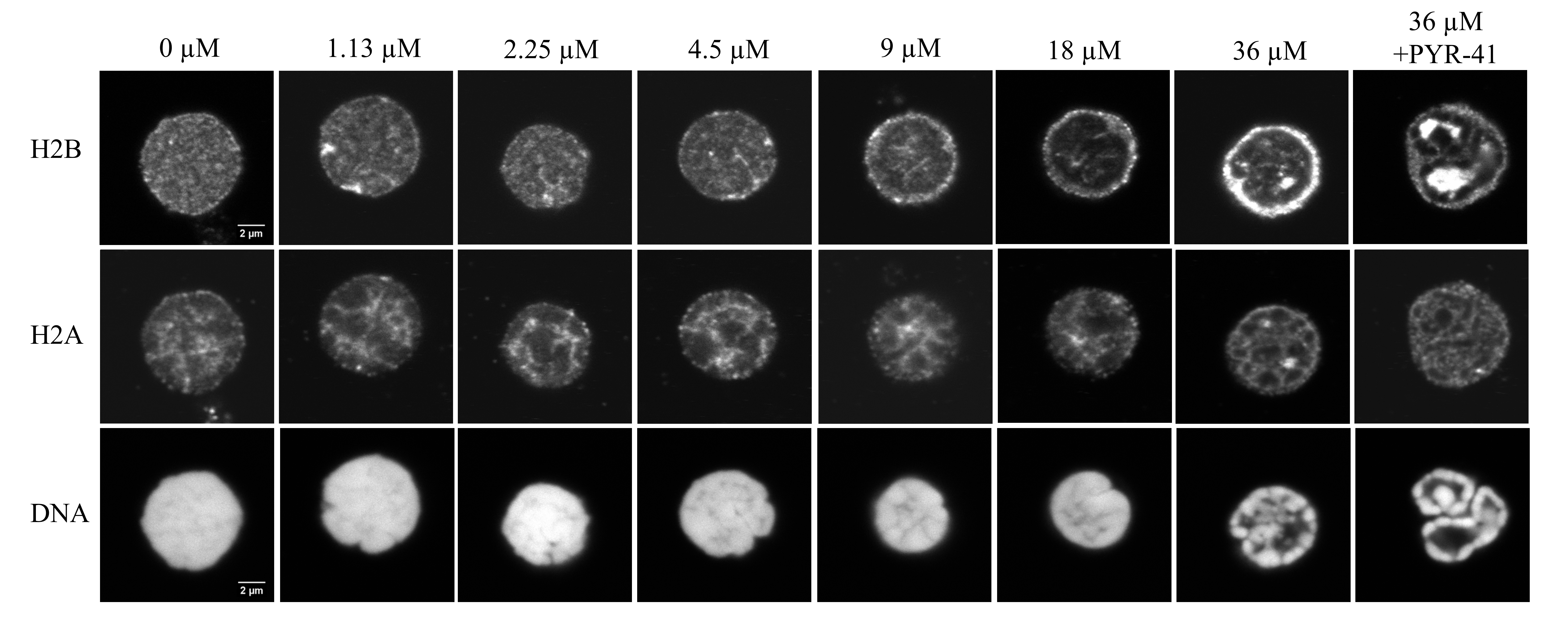

Supplement: S4 Fig — (TIF) [file pone.0231223.s005.tif]

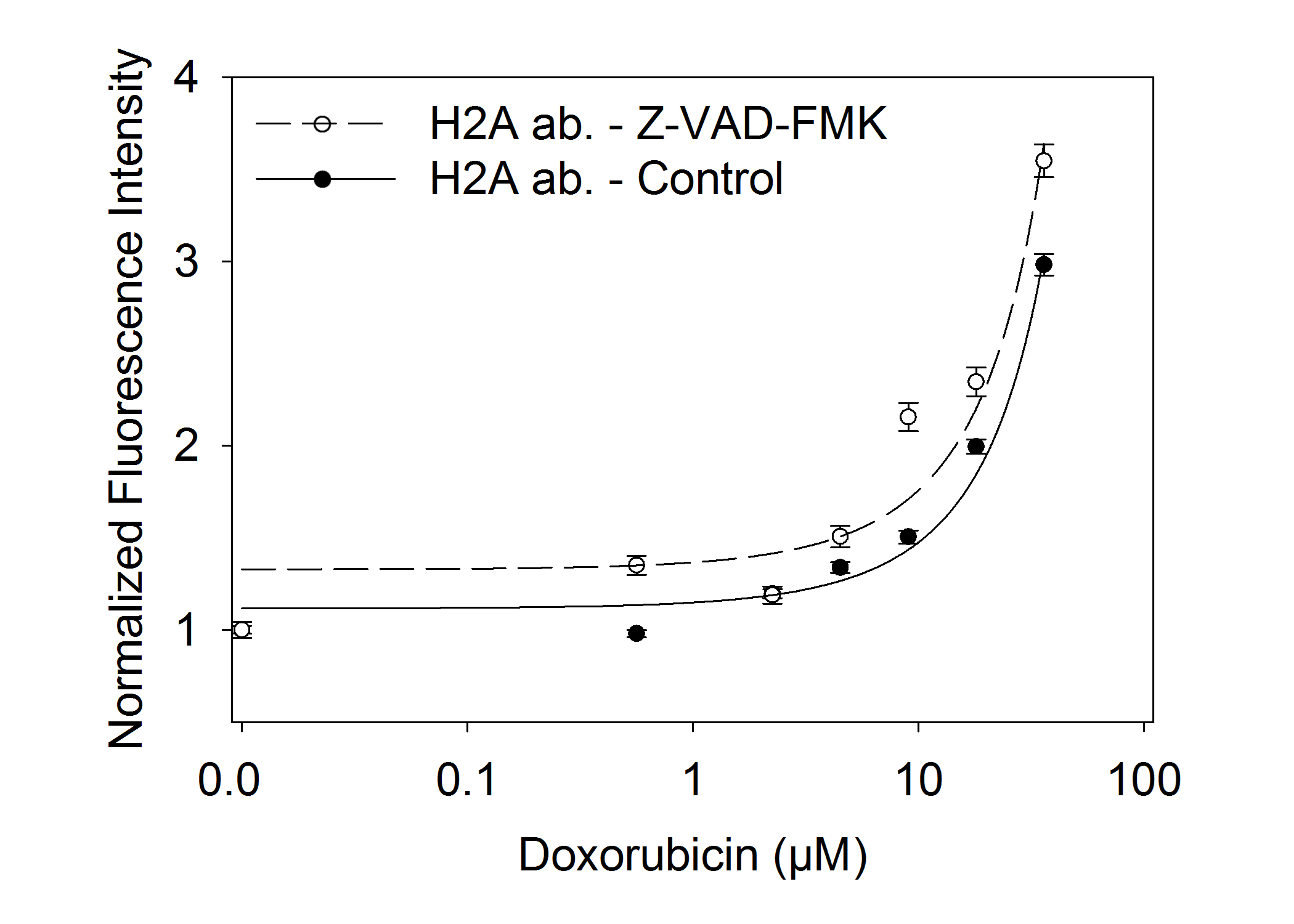

Supplement: S5 Fig — H2A levels after treatment with different concentrations of Dox alone (continuous line) or in the presence of 10 μM Z-VAD-FMK (caspase inhibitor) (dashed line). Fluorescence intensities were normalized to the intensity of untreated samples. Error bars show SEM values. (TIF) [file pone.0231223.s006.tif]

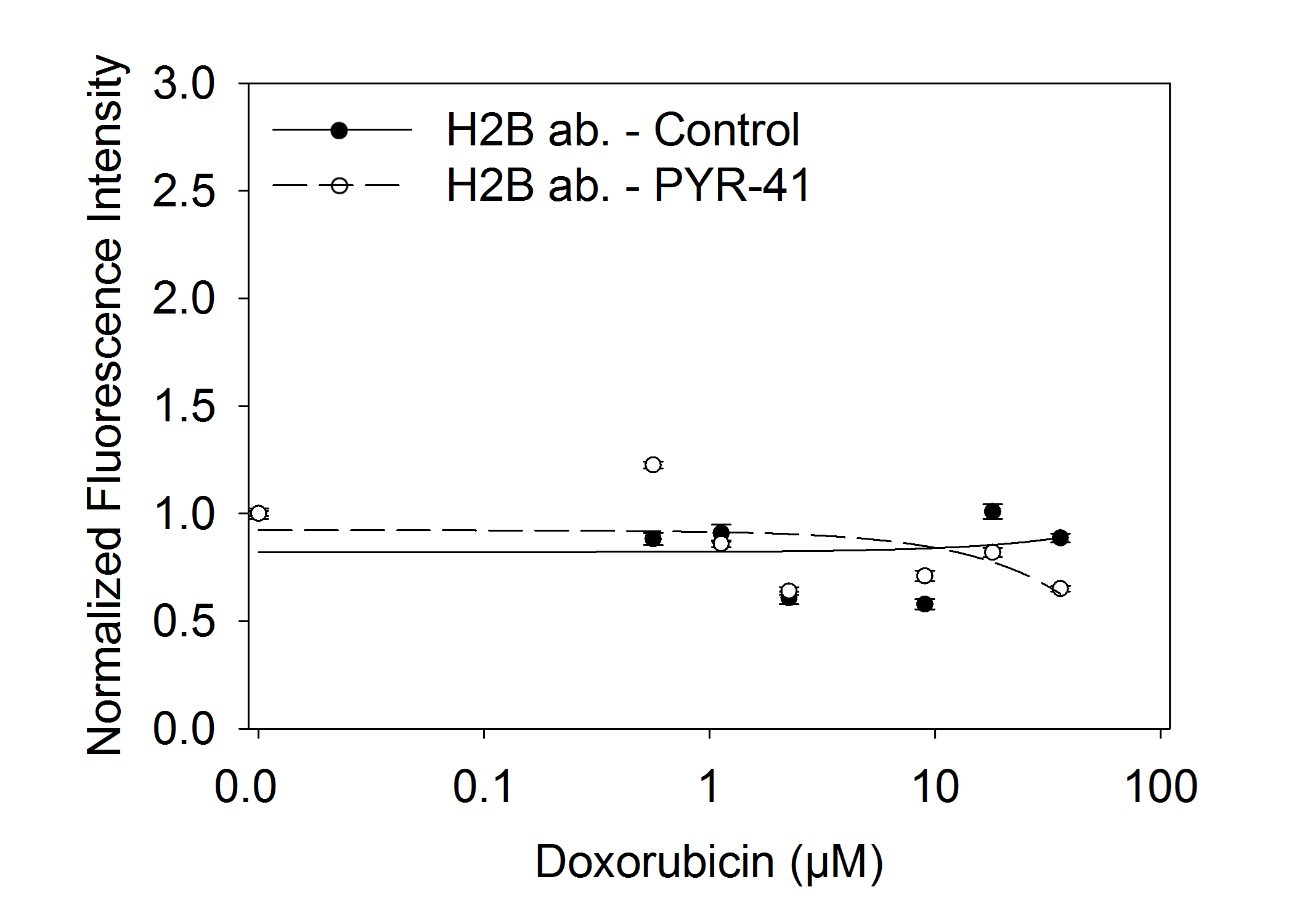

Supplement: S6 Fig — Intranuclear H2B levels after treating live cells with different concentrations of Dox alone (continuous line) and with Dox in the presence of 50 μM PYR-41 (dashed line). Fluorescence intensities were normalized to the intensity of untreated samples. Error bars show SEM values. (TIF) [file pone.0231223.s007.tif]

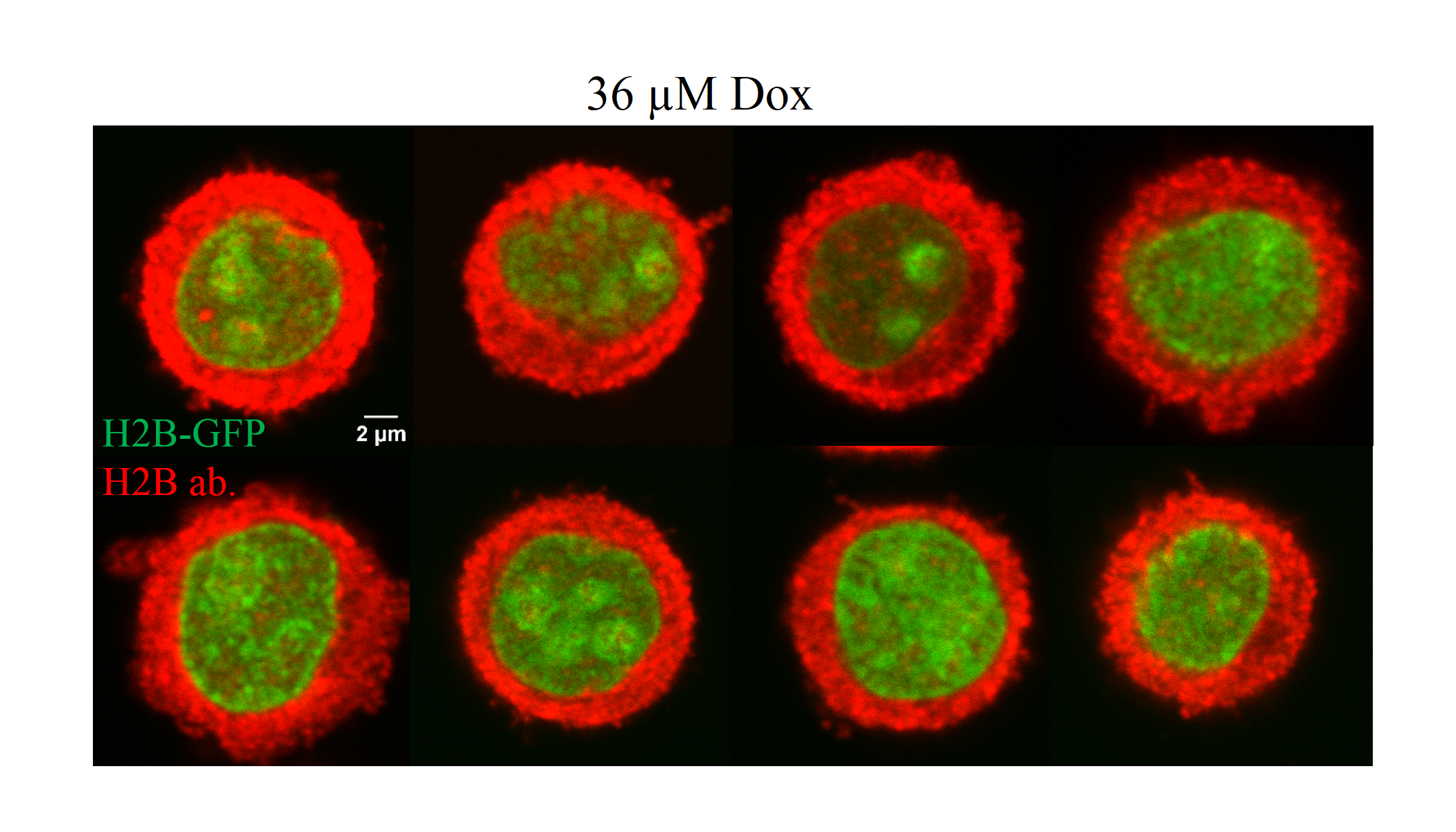

Supplement: S7 Fig — Representative confocal microscopic images of Dox treated H2B-GFP (green) expressor cells labeled with anti-H2B antibody (red). (TIF) [file pone.0231223.s008.tif]

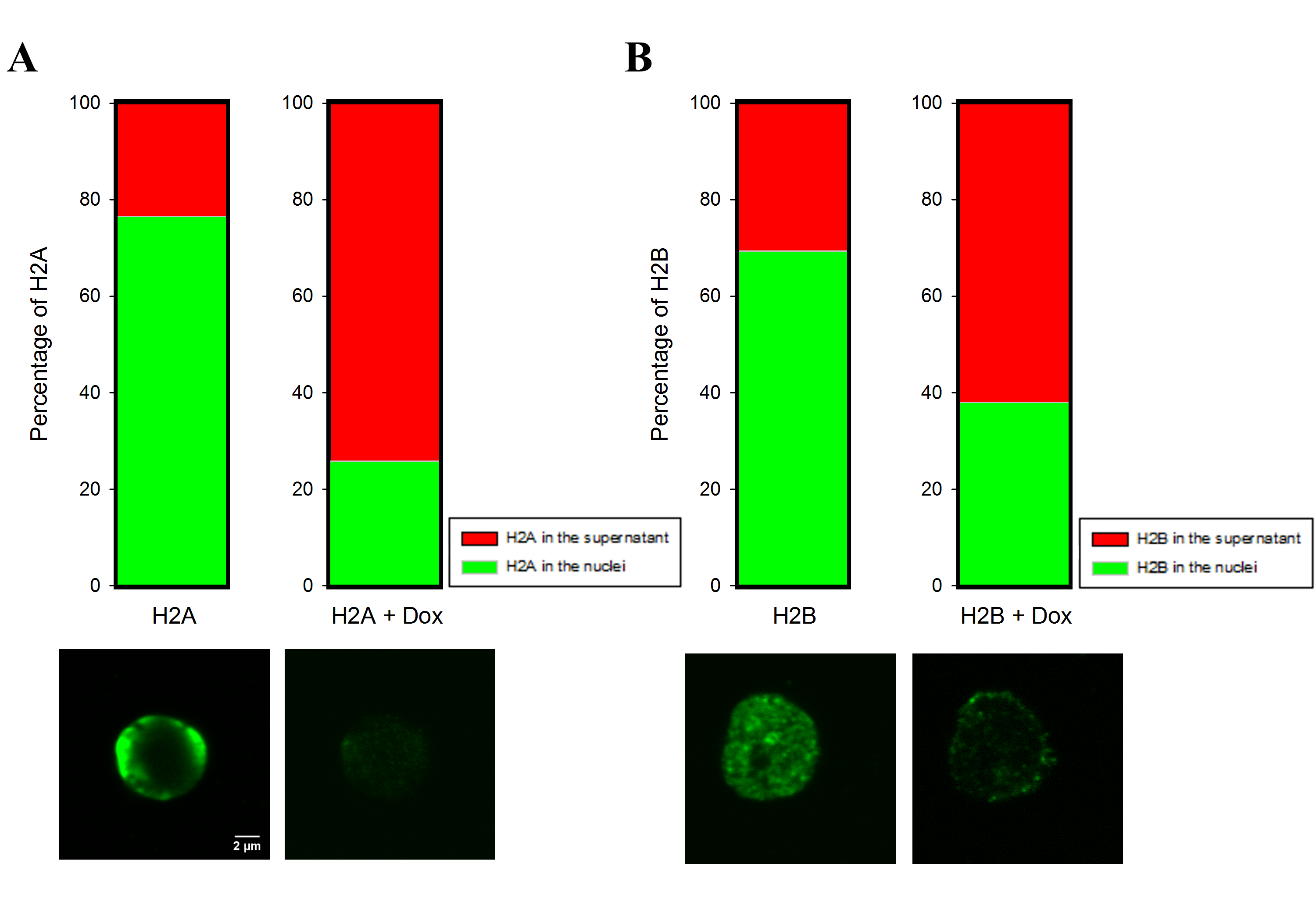

Supplement: S8 Fig — Fractions of H2A (panel A) and H2B (panel B) remaining in the nuclei or detected in the supernatant (indicated by green and red colors in the chart, respectively). The cell lysates were prepared without agarose-embedding, the histones were detected by MS in the supernatant and by LSC in the nuclei. The fractions shown in panels A and B were calculated as described in Materials and Methods. Representative microscopic images below show the histones remaining in the nuclei in these experiments. (TIF) [file pone.0231223.s009.tif]

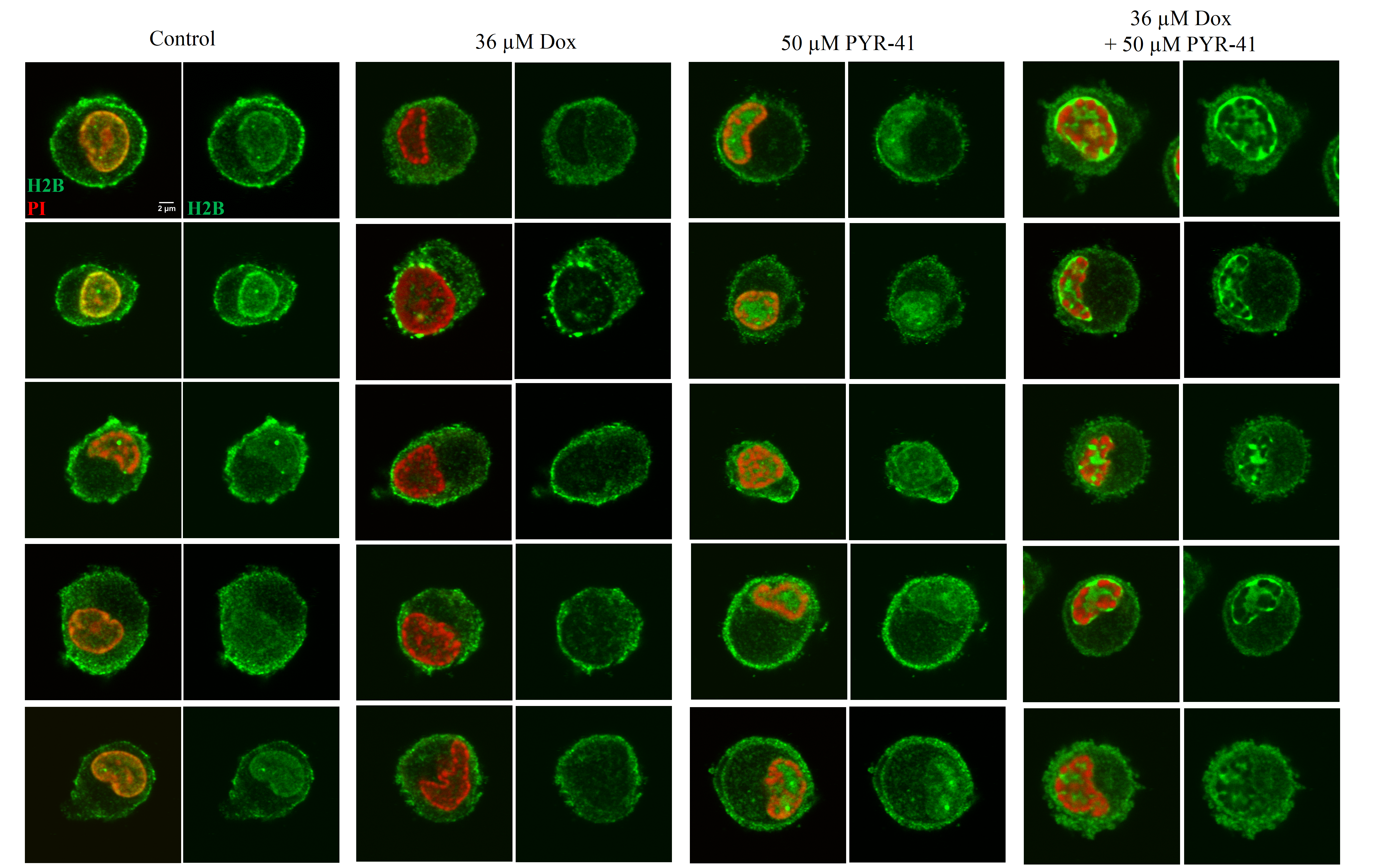

Supplement: S9 Fig — (TIF) [file pone.0231223.s010.tif]
